# Acetate attenuates kidney fibrosis in an oxidative stress‐dependent manner

**DOI:** 10.14814/phy2.15774

**Published:** 2023-07-18

**Authors:** Chiaki Kawabata, Yosuke Hirakawa, Reiko Inagi, Masaomi Nangaku

**Affiliations:** ^1^ Division of Nephrology and Endocrinology The University of Tokyo Graduate School of Medicine Tokyo Japan; ^2^ Division of Chronic Kidney Disease Pathophysiology The University of Tokyo Graduate School of Medicine Tokyo Japan

**Keywords:** acetate, chronic kidney disease, mitochondria, reactive oxygen species, short‐chain fatty acid

## Abstract

Short‐chain fatty acids (SCFAs) are the end products of the fermentation of dietary fibers by the intestinal microbiota and reported to exert positive effects on host physiology. Acetate is the most abundant SCFA in humans and is shown to improve acute kidney injury in a mouse model of ischemia–reperfusion injury. However, how SCFAs protect the kidney and whether SCFAs have a renoprotective effect in chronic kidney disease (CKD) models remain to be elucidated. We investigated whether acetate and other SCFAs could attenuate the kidney damage. In in vitro experiments, cell viability of acetate‐treated human kidney 2 (HK‐2) cells was significantly higher than that of vehicle‐treated in an oxidative stress model, and acetate reduced cellular reactive oxygen species (ROS) production. In mitochondrial analysis, the MitoSOX‐positive cell proportion decreased, and transcription of dynamin‐1‐like protein gene, a fission gene, was decreased by acetate treatment. In in vivo experiments in mice, acetate treatment significantly ameliorated fibrosis induced by unilateral ureteral obstruction, and the oxidative stress marker phosphorylated histone H2AX (γH2AX) was also reduced. Further, acetate treatment ameliorated dysmorphic mitochondria in the proximal tubules, and ROS and mitochondrial analyses suggested that acetate improved mitochondrial damage. Our findings indicate a renoprotective effect of acetate in CKD.

## Graphical abstract

Acetate treatment ameliorated dysmorphic mitochondria of proximal tubules in UUO mice. ROS and mitochondrial analyses suggested that acetate improved mitochondrial damage.

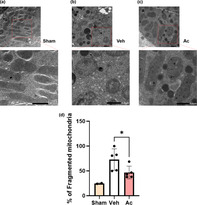

## 1 INTRODUCTION

Chronic kidney disease (CKD) increases the risk of death, cardiovascular disease (CVD) and hospitalization (Go et al., 2004). Although the prevalence varies according to ethnicity and socioeconomic status, rising prevalence and medical costs are worldwide problems. In addition, disability‐adjusted life years (DALYs) in patients with CKD, a measure that quantifies the overall burden of disease in terms of years of healthy life lost due to the disease, has increased by 62% over the last quarter‐century (Xie et al., 2018). Thus, prevention and inhibition of CKD progression are important.

Once kidney damage progresses to some extent, its progression is irreversible and leads to end‐stage kidney disease (ESKD). These changes occur regardless of primary kidney disease (Mimura & Nangaku, 2010; Nangaku, 2006). Kidney fibrosis is the final common pathological process in CKD, and its irreversibility causes unidirectional deterioration of kidney function. Therefore, the extent of fibrosis predicts kidney prognosis in CKD (Nath, 1992). Kidney fibrosis is characterized by excessive accumulation and deposition of extracellular matrix in the interstitial space (Arai & Yanagita, 2020). Increased intracellular levels of reactive oxygen species (ROS) can lead to tissue damage, including kidney fibrosis (Irazabal & Torres, 2020). Pharmaceutical interventions for kidney fibrosis are required to improve kidney survival. However, no existing drugs have been proven to ameliorate kidney fibrosis (Kalantar‐Zadeh et al., 2021). As kidney fibrosis is the final common pathological change in CKD, a drug that improves kidney fibrosis must exert independent effects on primary kidney disease and the extent of proteinuria. Sodium glucose 2 transporter inhibitor (SGLT2i) fulfills these requirements (Heerspink et al., 2020), but the mechanism by which SGLT2i protects the kidney is largely unknown. Therefore, it is important to discover novel therapeutic interventions that ameliorate kidney fibrosis.

Recently, short‐chain fatty acids (SCFAs) were reported to attenuate inflammatory and metabolic disorders. SCFAs are end products of the fermentation of dietary fibers by the intestinal microbiota. These are carboxylic acids containing one to six carbon atoms. The most abundant SCFA is acetate, followed by propionate and butyrate (Wong et al., 2006). Once SCFAs enter the bloodstream via monocarboxylate transporters (MCT) or by diffusion, they can exert cellular effect (Pluznick, 2016). SCFAs have been shown to ameliorate inflammatory bowel diseases (Venegas et al., 2019), insulin sensitivity (Müller et al., 2019), blood pressure and heart rate (Poll et al., 2021) and airway disease (Trompette et al., 2014). SCFAs in the bloodstream are absorbed by the kidneys via G‐protein‐coupled receptor (GPCR) or MCT. Among the four GPCRs, GPR41 and GPR43 are expressed in the whole kidney and renal artery, indicating that kidney utilizes acetate (Pluznick, 2016). The existence of these GPCRs in the kidney supports the notion that SCFAs are translocated into the kidney and act as biologically active substances. Indeed, acetate supplementation increases erythropoietin, a hormone produced in the kidney (Xu et al., 2014).

It has been reported that acetate improved acute kidney injury (AKI) (Andrade‐Oliveira et al., 2015), and that butyrate improved AKI and diabetic nephropathy (Huang et al., 2020; MacHado et al., 2012; Sun et al., 2013), but the mechanisms are not understood. Although few studies have examined the effect of SCFAs on the kidney, no studies to date have addressed their effect on kidney fibrosis. Previous reports have shown that acetate attenuates oxidative stress by reducing ROS and has antioxidant effects in hepatocytes (Yu et al., 2022) and β‐cells (Hu et al., 2020). Thus, we investigated whether acetate and other SCFAs attenuate ROS and kidney damage in CKD and how SCFAs affect kidney fibrosis.

## 2 MATERIALS AND METHODS

### 2.1 Cell culture

Cultured cells were maintained in a humidified incubator with 5% CO_2_. Hypoxic incubation was performed in a personal CO_2_/multi gas incubator APM‐30D (Astec).

HK‐2 cells (*Homo sapiens*, human kidney, male, CRL‐2190, ATCC, RRID:CVCL_0302), an immortalized proximal tubule epithelial cell line from a normal human adult kidney, were cultured in Dulbecco's modified Eagle's medium/nutrient mixture F‐12 Ham (DMEM/F12) (D8062, Sigma Aldrich) containing penicillin–streptomycin solution (15070063, Thermo Fisher Scientific) and 10% fetal bovine serum (FBS) (F7524, Sigma Aldrich) in a 10 cm culture dish. For passaging, HK‐2 cells were dissociated with trypsin (204‐16935, Wako Pure Chemical Industries) and centrifuged at 1000 rpm for 5 min.

### 2.2 Cell viability and cytotoxicity

Cell viability and cytotoxicity were evaluated using a Viability/Cytotoxicity Multiplex Assay Kit (346‐09271, Dojindo), according to the manufacturer's instructions. Briefly, HK‐2 cells (1 × 10^4^/well) were seeded in a 96‐well microplate (167008, Thermo Fisher Scientific). The following day, the medium was removed and SCFAs in DMEM/F12, HEPES, and no phenol red (11039021, Thermo Fisher Scientific) (200 μL/well) were added. The final concentrations of acetate, propionate, and butyrate used were 0.5 mM. They were incubated in 1% O_2_ for 16 h and then in normoxia for 3 h (Muratsu‐Ikeda et al., 2012), or in normoxia for 19 h.

Medium (100 μL) from each well was transferred to a new 96‐well microplate for the LDH assay; 100 μL of working solution was added to each well of a new plate and incubated at room temperature in the dark for 30 min, and 50 μL of stop solution was added. Absorbance was measured at 490 nm using a multimode plate reader (EnSpire™, PerkinElmer). LDH is a stable cytoplastic enzyme released into the medium through the damaged plasma membrane. The amount of formazan dye formed by LDH indicates cytotoxicity.

Ten microliters of CCK‐8 assay was added to each well of the remaining plate and incubated for 1 h at 37°C. Absorbance readings at 450 nm were recorded as described above. The amount of formazan dye generated by dehydrogenase activity in living cells is proportional to cell viability.

### 2.3 ROS assay

Intracellular ROS were detected using a 2′,7′‐dichlorofluorescin diacetate (DCFDA)/H2DCFDA—Cellular ROS Assay Kit (ab113851, Abcam) according to the manufacturer's instructions. DCFDA is a fluorogenic dye that measures cellular ROS activity within the cell. Briefly, HK‐2 cells (2.5 × 10^4^/well) were seeded in 96‐well Black/Clear Flat Bottom TC‐treated imaging Microplate (353219, Falcon). The following day, the medium was removed and SCFAs in DMEM/F12, HEPES, and no phenol red (100 μL/well) were added. The final concentrations of acetate, propionate, and butyrate used were 0.5 mM. The cells were incubated under normoxia for 19 h.

The cells were incubated with 20 μM DCFDA at 37°C for 45 min and fluorescence was measured (excitation 485 nm, emission 535 nm) using a multimode plate reader (EnSpire™).

### 2.4 Mitochondrial ROS

Mitochondrial ROS were detected using a MitoSOX™ Red Mitochondrial Superoxide Indicator for live‐cell imaging (M36008, Thermo Fisher Scientific) according to a flow‐cytometry‐based protocol (Yang et al., 2021). Briefly, HK‐2 cells (5 × 10^5^/dish) were seeded in a 10 cm culture dish. The following day, the medium was removed and SCFAs in DMEM/F12, HEPES, and no phenol red (10 mL/dish) were added. The final concentration of acetate was 0.5 mM, whereas the control did not contain SCFAs. The cells were incubated in 1% O_2_ for 16 h and then under normoxia for 3 h. The cells were dissociated with trypsin, washed in Hanks' balanced salt solution (HBSS), and resuspended in 2 μM MitoSOX red. After incubation for 30 min at 37°C, the cells were washed and resuspended in 1 mL of HBSS. The expression of MitoSOX in the PE channel was quantified using a CytoFLEX flow cytometer (Beckman Coulter).

### 2.5 Mito stress test

HK‐2 cells (1 × 10^4^/well) were seeded into a 96‐well microplate. The following day, the medium was removed and SCFAs in DMEM/F12, HEPES, and no phenol red (80 μL/well) were added. The final concentration of acetate was 0.5 mM, while the control did not contain SCFA. The cells were incubated in 1% O_2_ for 16 h and then under normoxia for 3 h. Metabolic flux was measured in real time using a Seahorse XFe96 Analyzer (Agilent) and a Mito Stress Test kit (103015‐100, Agilent). The concentrations of glucose, pyruvate, and glutamine in the culture media were 10, 1, and 2 mmol/L, respectively. Oligomycin was first injected and inhibited ATP synthase (complex V). The reduction in the oxygen consumption rate (OCR) is linked to ATP production. The second injection was carbonilcyanide *p*‐trifluoromethoxyphenylhydrazone (FCCP), which is an uncoupling reagent. The electron flow through the electron transport chain was uninhibited, and the OCR by complex IV reached a maximum. The third injection consisted of rotenone and antimycin A, a complex III inhibitor. They shut down the mitochondrial respiration.

### 2.6 Animal studies

Six‐week‐old male C57BL/6 mice (Jackson Laboratory) were randomly assigned to the vehicle‐only and acetate‐treated groups. We administered UUO to mice and PBS or 0.5 M acetate by intraperitoneal injection every other day from the day before UUO operation to Day 6 (10 mL/kg/day). The mice were sacrificed on Day 7. The mice were anesthetized with a mixture of medetomidine hydrochloride (0.3 mg/kg), butorphanol (5 mg/kg), and midazolam (4 mg/kg) before UUO operation and sacrifice. All animal experiments were approved by the ethics committee of the Graduate School of Medicine, The University of Tokyo (No.18‐P‐134), and performed in accordance with the guidelines established by the Committee on the Ethical Animal Care and Use of the University of Tokyo. The animal experiments were carried out in accordance with the ARRIVE guidelines.

### 2.7 Histology and immunohistochemistry

Formalin‐fixed and paraffin‐embedded sections of the kidney were stained with Masson's trichrome. Fibrosis was detected using Masson trichrome staining. Kidney cryosections embedded in Tissue‐Tek O.C.T compound (4583, Sakura Finetek Japan) (5 μm‐thick slices) were fixed with acetone for 10 min. After washing three times with Dulbecco's phosphate buffered saline (PBS), the cells were incubated with 5% bovine serum albumin (BSA) (A3059, Sigma Aldrich) for 30 min, and subsequently with serum‐free protein block (X0909, DAKO) for 10 min. The slices were then incubated overnight at 4°C with primary antibodies, anti‐gamma H2A.X antibody (1:5000 dilution, ab11174, Abcam). The sections were incubated with Alexa Fluor 594 anti‐rabbit IgG secondary antibody (1:200 dilution, R37119, Thermo Fisher Scientific) for 30 min at room temperature. Nuclear staining was performed using bisbenzimide H 33342 trihydrochloride (B2261, Sigma Aldrich) for each sample. Images were acquired using an inverted fluorescent microscope, BZ‐X710 (Keyence), with the following filters: Texas Red with an excitation wavelength (Ex) of 560/40 nm, emission wavelength (Em) of 630/75 nm, and DAPI (Ex: 360/40 nm, Em: 460/50 nm).

### 2.8 Western blotting

Kidney cortex homogenates were resuspended in radioimmunoprecipitation assay (RIPA) buffer containing 50 mM Tris–HCl (pH 8.0), 150 mM NaCl, 0.5% sodium deoxycholate, 0.1% sodium dodecyl sulfate (SDS), and 1.0% Triton X‐100 with a protease and phosphatase inhibitor cocktail (Thermo Fisher Scientific).

For western blotting, SDS sample buffer containing 60 mM Tris–HCl (pH 6.8), 2% SDS, 10% glycerol, 0.012% bromophenol blue, and 10 mM dithiothreitol (DTT) was added to the proteins. Proteins in SDS sample buffer were denatured by boiling at 95°C for 5 min. Proteins were separated by electrophoresis on 10% sodium dodecyl sulfate‐polyacrylamide gel. The proteins were then transferred onto a polyvinylidene fluoride membrane in a transfer buffer (48 mM Tris‐base buffer, 39 mM glycine, 0.04% SDS, and 20% methanol) using a Trans‐Blot® Turbo™ Transfer System (Bio‐Rad). Nonspecific protein binding was blocked with 5% skim milk in Tris‐buffered saline (pH 7.4) containing 0.05% Tween 20. The membranes were incubated with primary antibodies, rabbit polyclonal anti‐alpha smooth muscle (1:1000 dilution, ab5694, Abcam) or rabbit anti‐actin antibody (1:2000 dilution, A2066, Sigma‐Aldrich) at 4°C overnight, and then incubated at room temperature for 45 min with HRP‐conjugated goat anti‐rabbit IgG antibody (1:10,000 dilution, 170–6515, Bio‐Rad). The Pierce™ ECL Plus western blotting Substrate (32132, Thermo Fisher Scientific) was used for detection. The bands were observed using a Luminoimage Analyzer ImageQuant LAS 4000 (GE Healthcare). The intensity of bands was quantified using Multi Gauge version 3.2 software (Fujifilm) and normalized to actin.

### 2.9 Quantitative real‐time PCR (qRT‐PCR)

Total RNA was isolated using RNAiso Plus (9109, Takara) and reverse‐transcribed with RT Master Mix (Perfect Real Time) (RR036B, Takara Bio). Complementary DNA was subjected to quantitative real‐time PCR using the THUNDERBIRD SYBR qPCR Mix (QPS‐201, Toyobo) and a CFX Connect Real‐Time PCR System (Bio‐Rad). The transcript levels were normalized to β‐actin expression levels. qRT‐PCR was performed in triplicates using gene‐specific primers (Table 1). Outliers were removed using Grubbs' test.

**TABLE 1:** List of primers used in this study.

	Forward sequence	Reverse sequence
Human gene		
HMOX	CGGGCCAGCAACAAAGTG	AGTGTAAGGACCCATCGGAGAA
SOD1	AGGGCATCATCAATTTCGAG	TGCCTCTCTTCATCCTTTGG
SOD2	GGAAGCCATCAAACGTGACT	CTGATTTGGACAAGCAGCAA
CAT	TTTCCCAGGAAGATCCTGAC	ACCTTGGTGAGATCGAATGG
CYCS	AAGGGAGGCAAGCACAAGACTG	CTCCATCAGTGTATCCTCTCCC
FIS1	GTCCAAGAGCACGCAGTTTG	ATGCCTTTACGGATGTCATCATT
DRP1	CTGCCTCAAATCGTCGTAGTG	GAGGTCTCCGGGTGACAATTC
OPA1	TGTGAGGTCTGCCAGTCTTTA	TGTCCTTAATTGGGGTCGTTG
MFN2	CTCTCGATGCAACTCTATCGTC	TCCTGTACGTGTCTTCAAGGAA
ACTB	TCCCCCAACTTGAGATGTATGAAG	AACTGGTCTCAAGTCAGTGTACAGG
Mouse gene		
Fis1	GCCCCTGCTACTGGACCAT	CCCTGAAAGCCTCACACTAAGG
Drp1	GCGCTGATCCCGCGTCAT	CCGCACCCACTGTGTTGA
Opa1	TGGGCTGCAGAGGATGGT	CCTGATGTCACGGTGTTGATG
Mfn2	ACAGCCTCAGCCGACAGCAT	TGCCGAAGGAGCAGACCTT
Acta2	GTCCCAGACATCAGGGAGTAA	TCGGATACTTCAGCGTCAGGA
Col1a1	ACGCCATCAAGGTCTACTGC	ACTCGAACGGGAATCCATCG
Col3a1	TGACTGTCCCACGTAAGCAC	GAGGGCCATAGCTGAACTGA
Actb	AAGATCAAGATCATTGCTCCTCCTG	AAACGCAGCTCAGTAACAGTCC

### 2.10 Transmission electron microscopy

The kidney cortex was minced into 1 mm^3^ pieces, fixed in 2.5% glutaraldehyde solution in phosphate buffer (pH 7.4), postfixed with 1% osmium tetroxide, dehydrated, and embedded in epoxy resin. Ultrathin sections were stained with uranyl acetate and lead citrate, and examined under an electron microscope (JEM‐1400Flash, JEOL). Mitochondria (>2 μm in length) were considered filamentous. Fragmented mitochondria were defined as mitochondria with a length of <1 μm and spherical configuration (Zhan et al., 2015). Images were obtained in five random fields per kidney. Mitochondrial length was measured using the ImageJ software (NIH).

### 2.11 Statistical analysis

All data are expressed as mean ± standard error of the mean (SEM), with individual values in dot plots. Data were analyzed using one‐way or two‐way analysis of variance (ANOVA) for multiple comparisons or a Student's *t*‐test for comparison between the two groups. Statistical significance was set at *p* < 0.05. All statistical analyses were performed using GraphPad Prism version 9.3 (GraphPad Software). All data were measured in more than two technical replicates.

## 3 RESULTS

### 3.1 SCFAs influence HK‐2 cell viability under oxidative stress

**FIGURE 1:**
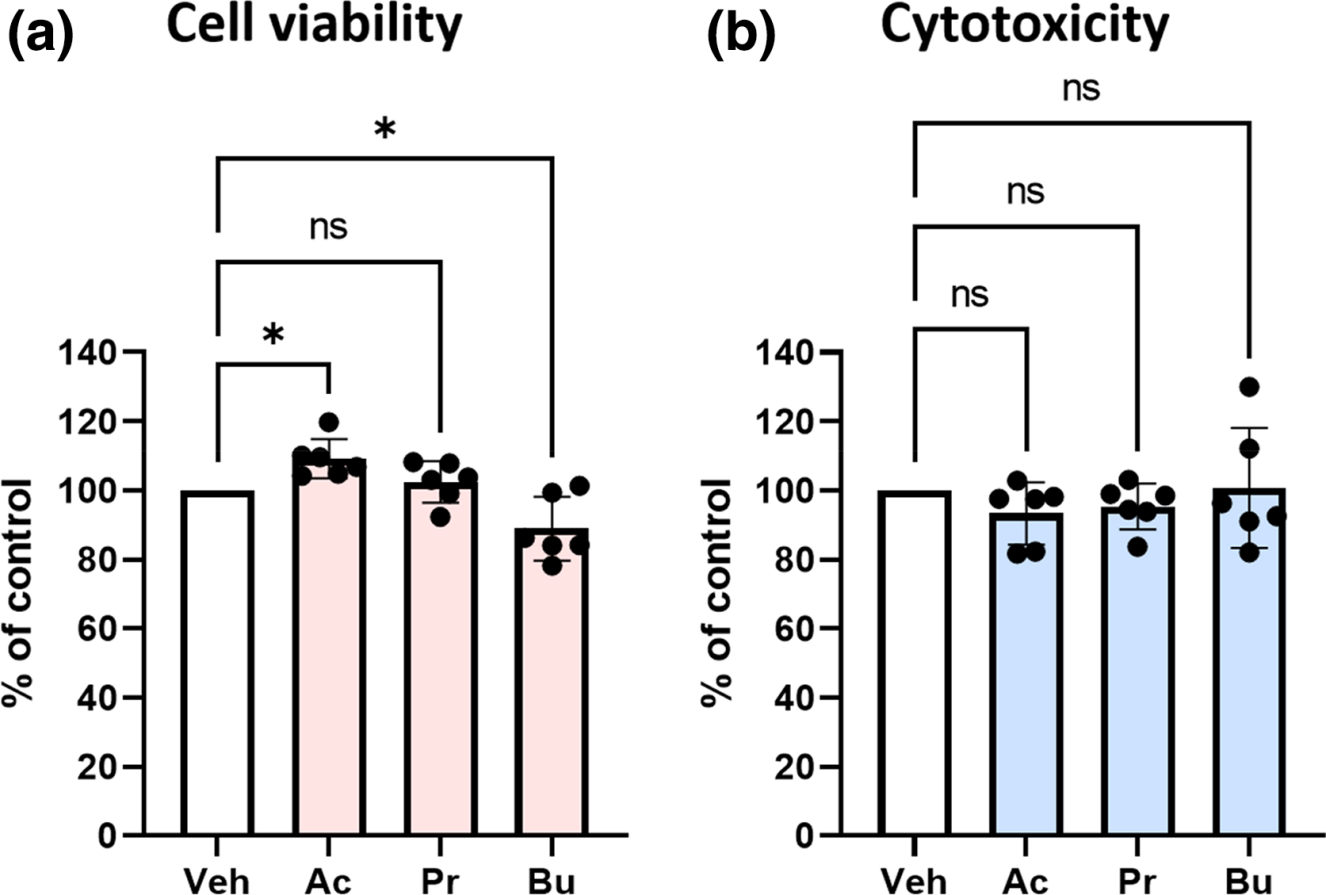
Effects of SCFAs on HK‐2 cell viability and cytotoxicity. (a) Cell viability and (b) cytotoxicity with 0.5 mM SCFA under hypoxia and reoxygenation (1% O_2_ for 16 h and normoxia for 3 h) (*n* = 5 in each group). Data are expressed as mean ± SEM. The statistical differences were quantified using one‐way ANOVA analysis with Dunnett's multiple comparisons test. *p < 0.05 compared with control. Ac, acetate; Bu, butyrate; Pr, propionate; Veh, vehicle.

To investigate the effect of SCFAs on HK‐2 cells, a proximal tubular cell line, cell viability was evaluated using the Cell Counting Kit‐8 (CCK‐8) assay, and cytotoxicity was evaluated using a lactate dehydrogenase (LDH) assay under 0.5 mM SCFA treatment. In hypoxia and reoxygenation, an oxidative stress model (Muratsu‐Ikeda et al., 2012), cell viability with acetate treatment was significantly higher than with vehicle (109.1 ± 2.8 vs. 100%, *p* < 0.05) (Figure 1a). The cytotoxicity of acetate treatment was lower, although the difference did not reach statistical significance (Figure 1b). In contrast, propionate and butyrate had no cell‐protective effects. Under normoxia, no significant differences were observed between the control and SCFAs (Figure S1a,b).

### 3.2 Acetate attenuates ROS production in HK‐2 cells

ROS levels were measured using DCFDA under normoxic conditions. Acetate treatment reduced ROS by 2.9 ± 0.9% (*p* < 0.05). To elucidate the mechanism of the antioxidant effect of acetate, we estimated the mRNA levels of the antioxidant genes heme oxygenase‐1 (HO‐1), superoxide dismutase (SOD)‐1, SOD‐2, and catalase under hypoxic conditions for 16 h. However, the acetate treatment did not alter the expression of these mRNAs, except for HO‐1 (Figure S2a–d). Although there was a statistically significant difference in HO‐1, we did not consider that the small difference lead to whole cellular protective effects. We also performed real‐time qPCR for cytochrome c (superoxide) under hypoxia for 16 h. No significant difference was observed between the vehicle and SCFAs (Figure S2e), as well as the other results of the qPCR.

### 3.3 Acetate has a partial beneficial effect on HK‐2 cell mitochondria under oxidative stress

**FIGURE 2:**
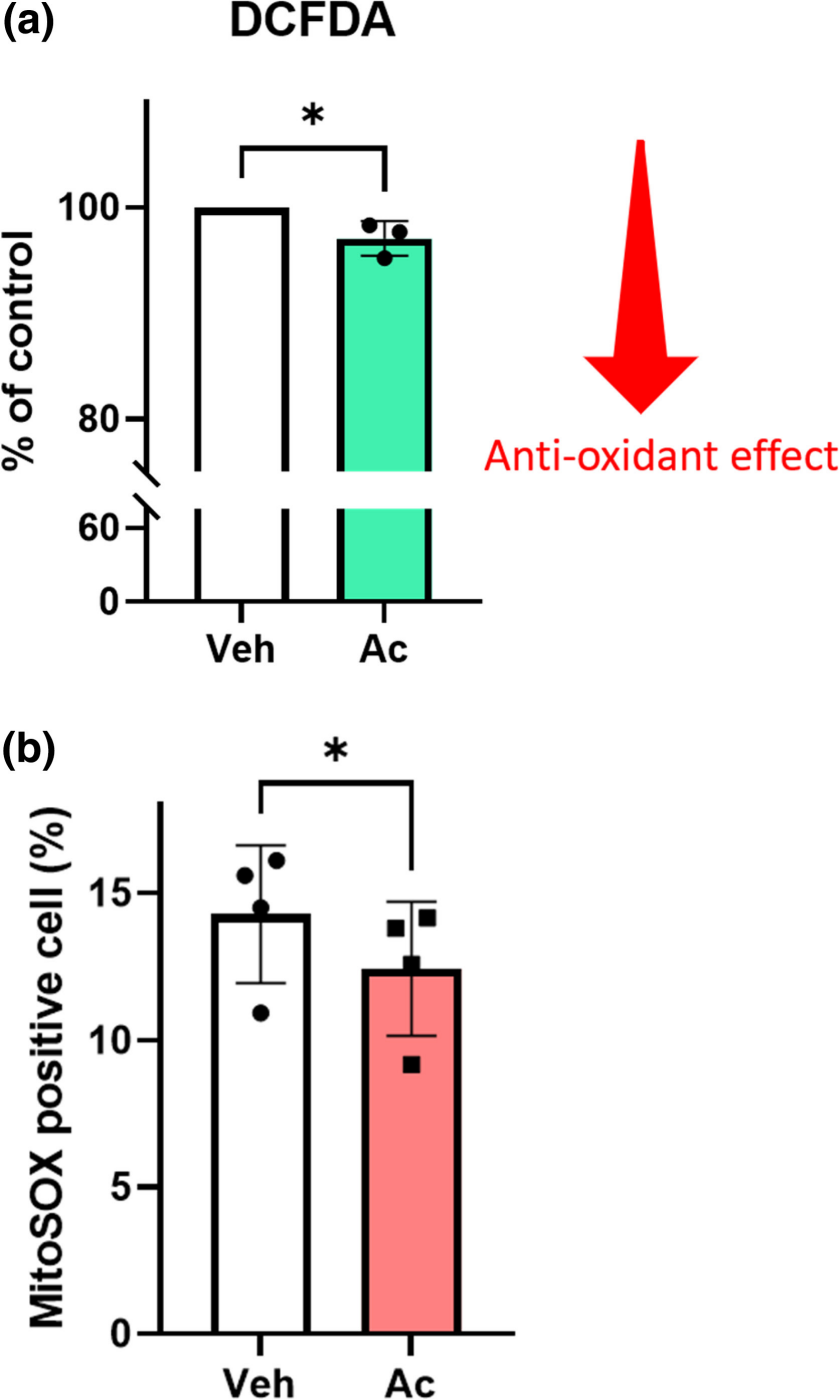
Acetate reduced ROS and mitochondrial ROS in HK‐2 cells. (a) Effects of SCFAs on ROS production under normoxia 19 h (*n* = 3 in each group). Quercetin is one of the antioxidant flavonoids and positive control. (b) MitoSOX positive cell proportion significantly decreased under hypoxia and reoxygenation (1% O_2_ for 16 h and normoxia for 3 h) (*n* = 3 in each group). Data were taken in 10,000 events per sample. Data are expressed as mean ± SEM. *p < 0.05 compared with control (*t* test). Ac, acetate; ROS, reactive oxygen species; SCFAs, short‐chain fatty acids; Veh, vehicle.

Because mitochondria are a major source of ROS (Dan Dunn et al., 2015), we speculated that acetate supplementation might change mitochondrial conditions and functions. Therefore, we evaluated mitochondrial ROS levels under hypoxia and reoxygenation. In the acetate treatment group, the MitoSOX‐positive cell rate significantly decreased, indicating that acetate supplementation decreased not only total cellular ROS but also mitochondrial ROS (Figure 2b).

To investigate how acetate affects cell metabolism, we conducted a mitochondrial stress test under hypoxia and reoxygenation conditions using a flux analyzer. Mitochondrial respiration was evaluated based on basal respiration, ATP production, spare respiration, and maximal respiration. Despite the mitochondrial ROS production gap, no significant difference was observed in mitochondrial respiration between the vehicle and acetate groups (Figure S3a–e). Mitochondrial homeostasis requires a subtle balance between mitochondrial energetics and dynamics to maintain proper function (Bhargava & Schnellmann, 2017). Therefore, we focused on the mitochondrial morphology.

### 3.4 Acetate partially affects mitochondrial fission/fusion gene expression under oxidative stress in HK‐2 cells

**FIGURE 3:**
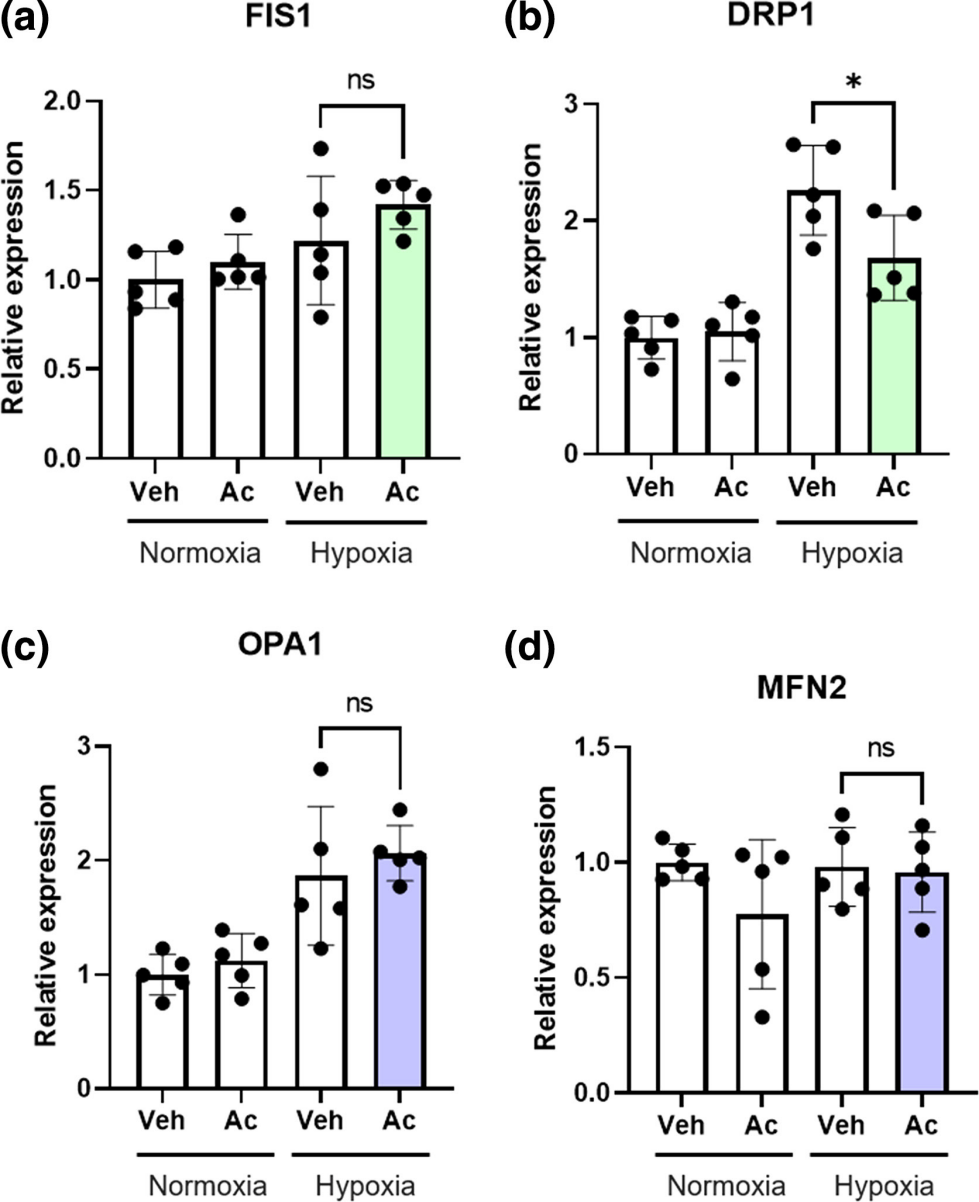
Acetate decreased DRP‐1 mRNA expression under oxidative stress in HK‐2 cells. (a–d) Effects of acetate on fission/fusion gene expression under normoxia and hypoxia for 16 h (*n* = 5 in each group). Data are expressed as mean ± SEM. The statistical differences were quantified using two‐way ANOVA analysis with Sidak's multiple comparisons test. *p < 0.05 compared with control. Ac, acetate; DRP‐1, dynamin‐1‐like protein; FIS1, fission protein 1; MFN2, mitofusin 2; OPA1, optic atrophy 1; Veh, vehicle.

To elucidate the tubular cell protective and mitochondrial protective mechanisms of acetate, transcription of mRNA of fission genes, fission protein 1 (FIS1) and dynamin‐1‐like protein (DRP‐1), and fusion genes, optic atrophy 1 (OPA1) and mitofusin 2 (MFN2), were examined. The mRNA expression of DRP‐1 was increased by hypoxic stimulation, and was significantly suppressed by acetate treatment (Figure 3b). The effect of acetate canceling out of hypoxia could not be observed in three genes other than DRP‐1 (Figure 3a,c,d). Previous studies have shown upregulated DRP‐1 and fragmented mitochondria in CKD caused by diabetic kidney disease (DKD) (Jiang et al., 2019; Zhan et al., 2015). In contrast, acetate supplementation reduced DRP‐1 expression under oxidative stress conditions.

### 3.5 Acetate treatment ameliorates kidney fibrosis in unilateral ureteral obstruction (UUO) mice

**FIGURE 4:**
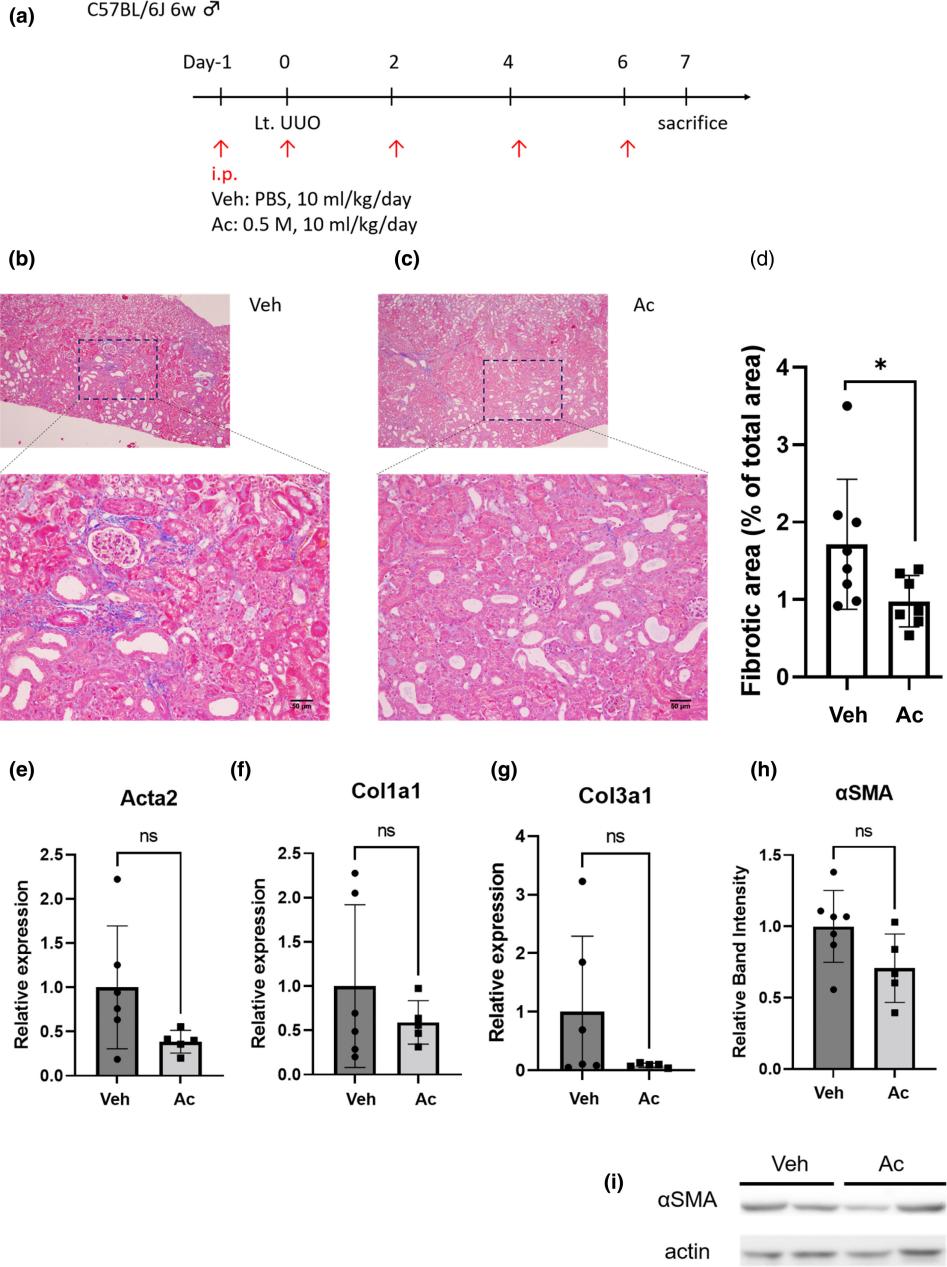
Acetate treatment ameliorated kidney fibrosis in UUO mice. (a) Schematic diagrams of the vivo experimental procedure (Veh, *n* = 8; Ac, *n* = 7). (b–d) Histological analysis of tubulointerstitial fibrosis by Masson trichrome staining. Data are average of five random area per sample. (e–g) Effects of acetate on transcription of mRNA of fibrosis marker. (h) Analysis of western blotting for αSMA protein. (i) Representative image of western blotting for αSMA. Data are expressed as mean ± SEM. *p < 0.05 compared with control (*t* test). Ac, acetate; Acta2, actin alpha 2; αSMA, α‐smooth muscle actin; Col1a1, Collagen 1; Col3a1, Collagen 3; UUO, unilateral ureteral obstruction; Veh, vehicle.

To investigate whether acetate attenuates kidney fibrosis in vivo via tubule protection, mice were subjected to UUO and treated with acetate via intraperitoneal injection. Acetate treatment diminished the area of kidney fibrosis compared with phosphate‐buffered saline (PBS) treatment (1.0% vs. 1.7%, *p* < 0.05) (Figure 4a–d). Although the difference did not reach statistical significance, it had a tendency to reduce transcription of mRNA of fibrosis markers αSMA, collagen 1, and collagen 3 by acetate treatment (Figure 4e–g). αSMA protein synthesis was also suppressed by acetate treatment (Figure 4h,i).

### 3.6 Acetate treatment ameliorates double‐strand DNA damage by oxidative stress in UUO mice

**FIGURE 5:**
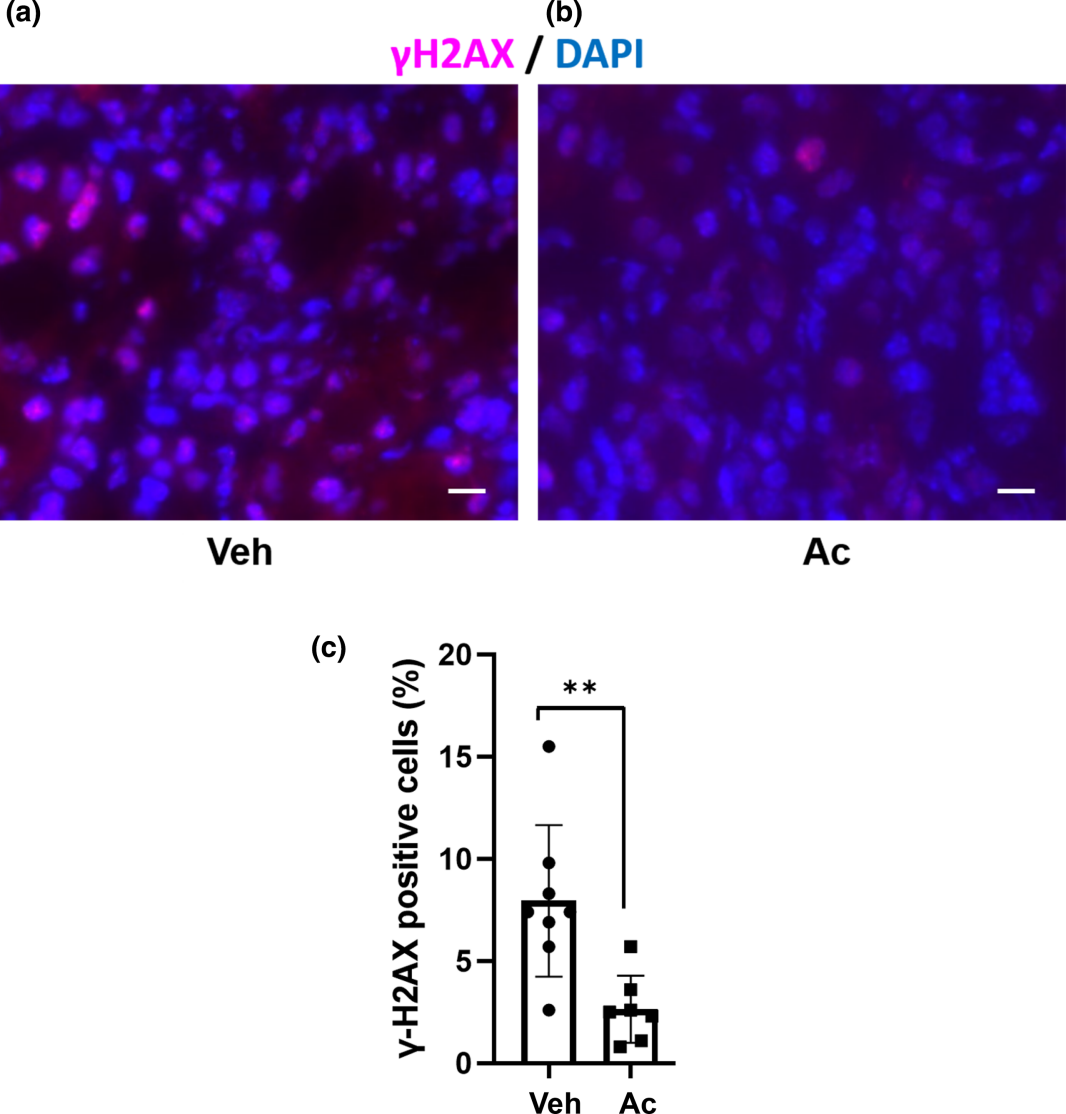
Acetate treatment reduced DNA damage by oxidative stress in UUO mice. (a–c) Immunofluorescence staining for γH2AX in control and acetate treatment (Veh, *n* = 8; Ac, *n* = 7). Data are average of nine random area per sample. Data are expressed as mean ± SEM. **p < 0.01 compared with control (*t* test). Ac, acetate; DAPI, 4′,6‐diamidino‐2‐phenylindole; γH2AX, phosphorylated histone H2AX; Veh, vehicle; scale bars, 10 μm.

To determine whether this amelioration of fibrosis occurred via tubule protection, we assessed oxidative stress in the tubules. DNA damage, detected by immunohistochemistry of phosphorylated histone H2AX (γH2AX), reflects oxidative stress. Therefore, γH2AX was used as a surrogate marker for oxidative stress‐induced cell damage. Acetate treatment reduced the proportion of γH2AX positive cells by 5.3 ± 1.5% (*p* < 0.05) in UUO mice (Figure 5a–c). In the low power field of the staining, the area thought to be the glomerulus was not stained with γH2AX (arrow) (Figure S4). We thought that the area stained with γH2AX was mainly the tubules.

### 3.7 Acetate treatment alters mitochondrial morphology in proximal tubules in UUO mice

Considering the results of the hypoxia and reoxygenation model in HK‐2 cells, these effects of acetate appeared to be mediated by the effect of acetate on mitochondria. To investigate whether mitochondrial fission/fusion genes were affected by acetate, transcription of fission genes Fis1 and Drp‐1, and fusion genes Opa1 and Mfn2, was examined. However, no significant difference was observed between the vehicle and acetate treatment groups in UUO mice (Figure S5a–d). We considered this to be likely because we evaluated the whole cortex, not the tubular cells only.

**FIGURE 6:**
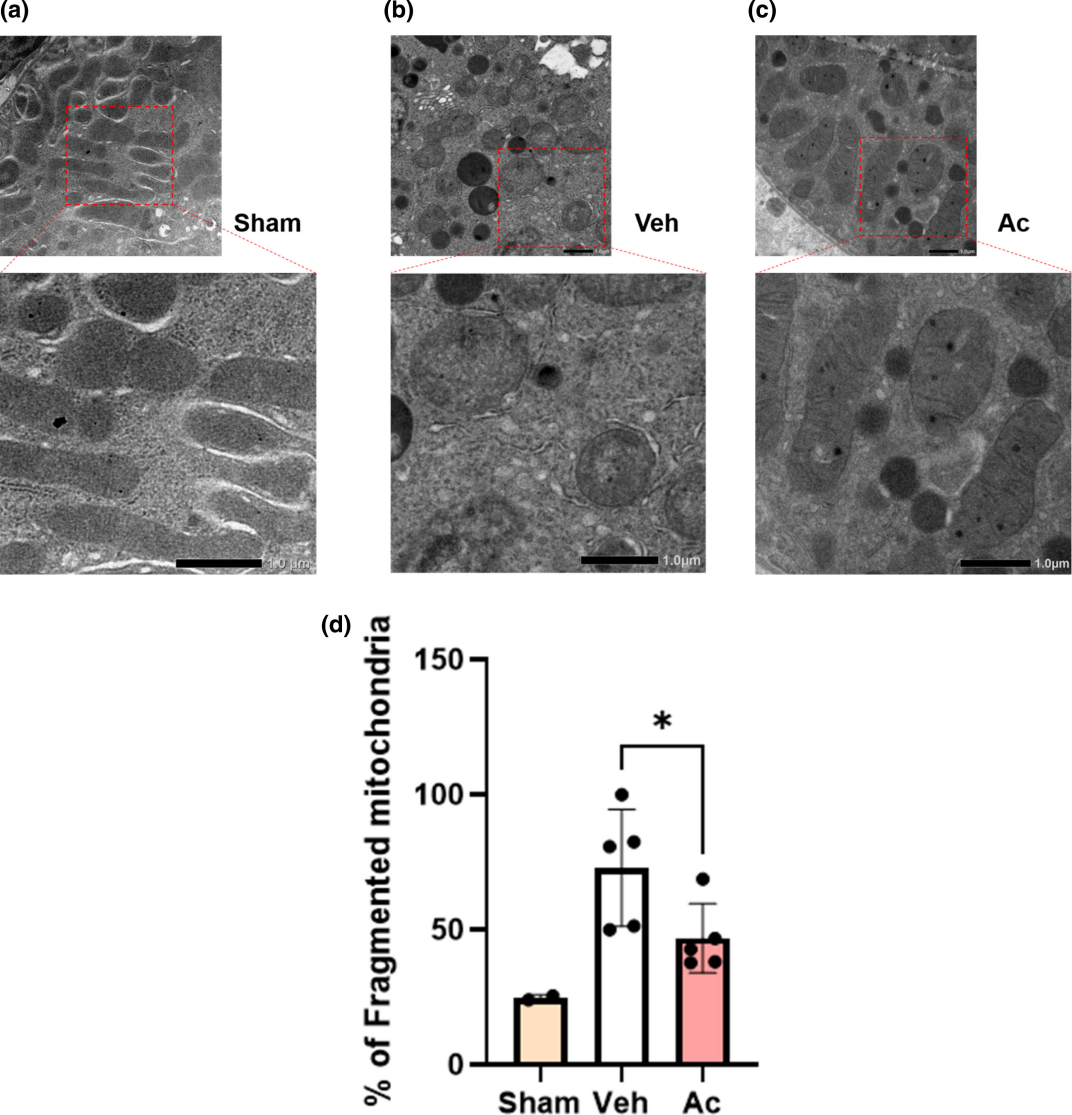
Acetate treatment ameliorated dysmorphic mitochondria of proximal tubules in UUO mice. (a–d) Histological analysis of mitochondrial morphology by electron microcopy (Sham, *n* = 3; Veh, *n* = 5; Ac, *n* = 5). Data are average of five random area per sample. Fragmented mitochondria were defined as mitochondria with length <1 μm and spherical configuration. Data are expressed as mean ± SEM. *p < 0.05 compared with control (*t* test). Ac, acetate; Veh, vehicle.

Therefore, we evaluated the mitochondrial morphology in the tubules using electron microscopy. In the sham group, the majority of proximal tubular cells had elongated mitochondria (Figure 6a). In the PBS‐treated UUO group (Veh), mitochondrial morphology changed to spheres or shot rods and cristolysis, which should reflect mitochondrial fission (Figure 6b). In contrast, in the acetate‐treated UUO group (Ac), the dysmorphic mitochondria were partially ameliorated (Figure 6c). The proportion of fragmented mitochondria was reduced by acetate compared with PBS and control, by 26.1 ± 11.2% (*p* < 0.05) (Figure 6d).

## 4 DISCUSSION

In this study, we demonstrated that acetate increased cell viability under oxidative stress, reduced ROS and mitochondrial ROS production, and decreased DRP‐1 mRNA expression under oxidative stress in HK‐2 cells. We also revealed that acetate ameliorated kidney fibrosis, DNA damage caused by oxidative stress, and mitochondrial dysmorphology in UUO mice.

We conducted these experiments using hypoxia and reoxygenation models in vitro and a UUO mouse model in vivo as an oxidative stress model. The gap between the in vitro and in vivo models should be explained. The UUO model was selected as we focused on kidney fibrosis. In addition, multiple signaling pathways can be activated during fibrosis, and one of the most important signaling pathways is angiotensin II (Ang II)/ROS (Aranda‐Rivera et al., 2021; Liu et al., 2017; Panizo et al., 2021; Sachse & Wolf, 2007). In the UUO model, increased hydrostatic pressure due to ureteral obstruction activates renin‐angiotensin system (RAS), which leads to the production of Ang II. Ang II activates nicotinamide adenine dinucleotide phosphate (NADPH) oxidases (NOXs), which produce ROS. ROS play an important role as secondary messengers in cellular signaling (Irazabal & Torres, 2020). In contrast, in vitro, we chose the hypoxia and reoxygenation model (Li & Jackson, 2002; Muratsu‐Ikeda et al., 2012), which is a representative oxidative stress model. Oxidative stress plays a crucial role in the damage induced by ischemia–reperfusion or hypoxia–reoxygenation. In vitro, a model with hypoxia in 1% O_2_ for 16 h and normoxia for 3 h was already established (Muratsu‐Ikeda et al., 2012). The degree of cell death was thought to reflect the effect of ROS in a hypoxia–reoxygenation model. We also performed the experiments using a transforming growth factor beta 1 (TGF‐β1)‐stimulated and H_2_O_2_‐induced oxidative stress models. However, the TGF‐β1‐stimulated model could not induce cytotoxicity. In contrast, the effects of acetate in the H_2_O_2_ model showed strong cytotoxicity. Acetate presumably reacted directly with H_2_O_2_ and became peracetic acid, which is used as a germicidal agent. Thus, we adopted only hypoxia and reoxygenation models.

SCFAs have often been treated as a group (Tahara et al., 2018; Yang et al., 2018). However, in this study, only acetate treatment consistently yielded positive results for cell viability and ROS production. Therefore, we focused only on acetate among the SCFAs after these experiments. One of the major roles of acetate is as an energetic source in the tricarboxylic acid (TCA) cycle. Mitochondrial acetate is converted to acetyl coenzyme A (acetyl‐CoA) by acyl‐CoA short‐chain synthetase (ACSS)1, after which acetyl‐CoA enters the TCA cycle. Another role is histone acetylation in the nucleus. Cytosol acetate is converted to acetyl‐CoA by ACSS2, and then acetyl‐CoA translocates to the nucleus and promotes gene transcription via histone acetylation (Martínez‐Reyes & Chandel, 2020; Pietrocola et al., 2015; Shi & Tu, 2015). We chose 0.5 mM SCFA with reference to the previous report (Xu et al., 2014). Additionally, acetate level in plasma is between 0.1 and 10 mM (Pluznick, 2016). Based on these findings, we considered that 0.5 mM is a physiological and appropriate concentration.

Recently, Hu et al. (2020) showed that butyrate inhibited the SCFA receptor GRP41 and NO generation more strongly than acetate in β‐cells. Dong et al. (2017) indicated that butyrate ameliorated diabetic nephropathy by inhibiting histone deacetylase (HDAC) activity. Based on these prior studies, we thought that acetate might have other protective effects.

Thus, we focused on the main origin of free radicals, mitochondria (Dan Dunn et al., 2015). As expected, mitochondrial ROS production was reduced by acetate treatment. Mitochondrial homeostasis requires a subtle balance between mitochondrial energetics and dynamics to maintain proper function (Bhargava & Schnellmann, 2017). We focused on mitochondrial morphology because no significant difference was observed in mitochondrial respiration between the control and acetate groups. Mitochondrial morphological dynamics are determined by the balance between fission and fusion in response to various stresses, and mitochondria continuously undergo fission and fusion (Youle & van der Bliek, 2012). Regarding the transcription of mitochondrial fission/fusion genes, the expression of only DRP‐1, a fission gene, was significantly increased by hypoxic stimulation, and was suppressed by acetate. Perry et al. (2018) reported that proximal tubule deletion of Drp‐1 prevented AKI and progression to fibrosis. Aranda‐Rivera et al. (2021) reported that ROS upregulates mitochondrial fission genes and downregulates fusion genes, suggesting that the reduction of ROS by acetate was the primary contributor to the decreased expression of DRP‐1. Compared to the previous study that showed both mitochondrial respiration and fission/fusion genes changes (Hu et al., 2020), in this study, acetate partially affected mitochondrial function of HK‐2 cells. We could not reveal the interaction between mitochondrial respiration and ROS, but we clarified that ROS reduction and DRP‐1 downregulation by acetate supplementation led to the rescue of mitochondrial dysfunction.

In vivo, acetate treatment ameliorated kidney fibrosis and DNA damage caused by oxidative stress in UUO mice. In addition, acetate improved the dysmorphic mitochondria. This morphological change corresponded to the decreased expression of DRP‐1 in vitro. However, no change in Drp‐1 expression was observed in vivo. This discrepancy could be explained by the difference between in vitro and in vivo results. Animal samples contain various contaminants and are unsuitable for the evaluation of the mitochondrial fraction. Another explanation is that Youle and van der Bliek (2012) proposed that the activity of DRP‐1 results from an equilibrium between the phosphorylation of DRP‐1 at serine 616, fission, and at serine 637, fusion. Evaluation of DRP‐1 phosphorylation in the mitochondrial fraction of cultured tubular cells is required in the future. The previous reports revealed elevated plasma acetate levels peaking at 10 min after injection and were near baseline at 60 min by intraperitoneal injection of acetate (500 mg/kg), which is almost the same amount as our study (Frost et al., 2014; Shubitowski et al., 2019). Although the half‐life of SCFAs is shorter than our dosing schedule, we referred to the schedule of the other previous report that the intraperitoneal injection of 0.5 M and 15 mL/kg SCFA solution exerted bioprotective effects thrice‐weekly (Monday, Wednesday, Friday) (Xu et al., 2014). We could not assess whether pre‐ and post‐injury SCFAs administration or only post‐injury administration was more effective. However, according to the previous report that SCFAs were administered both 30 min pre‐injury before ischemia and at the moment of reperfusion in the ischemia–reperfusion injury model (Andrade‐Oliveira et al., 2015), pretreatment seemed unnecessary for kidney protection.

One of the strengths of this study is that it revealed the effects of acetate on CKD. To the best of our knowledge, this is the first study to investigate the pathophysiology of acetate in CKD. The previous study only showed that the production of intestinal SCFAs decreased due to dysbiosis in CKD (Mishima et al., 2017). In addition, we demonstrated for the first time that acetate plays both tubular cell‐protective and renoprotective roles via mitochondrial mechanisms. Pluznick (2016) reported that increasing microbial SCFAs production using a high‐fiber diet or decreasing microbial SCFAs using a low‐fiber diet caused drastic changes in microbial diversity and serum SCFAs (Wagenaar et al., 2021). Increasing serum acetate levels by a high‐fiber diet may have a renoprotective effect. Several drugs are known to influence acetate levels. The most promising drug class to ameliorate kidney fibrosis is sodium‐glucose cotransporter‐2 inhibitors (SGLT2i), considering the results of clinical trials (Heerspink et al., 2020; Perkovic et al., 2019). SGLT2i influences gut microbiota and increases SCFA content, including acetate in the gut (Mishima et al., 2018). Therefore, the tubular protective effect of acetate revealed in this study can partly explain the renoprotective effects of SGLT2i. The previous study demonstrated that acetate ameliorated diabetes‐induced nephrotoxicity, including non‐tubular lineage, such as Bowman's space and congested glomeruli due to pro‐inflammatory mediators (Olaniyi et al., 2021). In the UUO model, acetate might also affect other kidney cells, thereby leading to suppression of fibrosis.

This study has several limitations. First, we did not show the results using primary cells. It is desirable to use primary cells, especially when evaluating cell metabolism. Although HK‐2 cells are immortalized, the in vitro results were consistent with the in vivo results. Therefore, we used the results obtained using HK‐2 cells. Second, we did not measure blood pH levels, and thus could not completely exclude the influence of pH on the kidney in in vivo experiments. On the contrary, we confirmed a constant pH of the SCFA‐included medium in the range of 7.15–7.19 by adding 4‐(2‐Hydroxyethyl) piperazine‐1‐ethanesulfonic acid (HEPES). Third, we did not examine histone acetylation in the present study. Therefore, the involvement of histone acetylation cannot be ruled out. Fourth, in the in vivo experiments, we did not use proximal tubules but the kidney cortex, which may explain the dissociation of mitochondrial fission/fusion gene expression between cultured tubular cells and the kidney.

## 5 CONCLUSIONS

In summary, we demonstrated that acetate had cell protective effects and that acetate ameliorated kidney fibrosis under oxidative stress condition. According to ROS and mitochondrial analysis, it was suggested that acetate improved damaged mitochondria. Our findings indicate renoprotective effect of acetate on CKD.

## AUTHOR CONTRIBUTIONS

C. K. and Y. H. designed the study, performed the experiments, and analyzed the data. C.K. wrote the manuscript. Y. H., R. I., and M. N. provided conceptual advice and edited the manuscript.

## FUNDING INFORMATION

This work was supported by JSPS KAKENHI Grant Number 22K08348 and partially performed in collaboration with Mitsubishi Tanabe Pharma Corporation under the Kidney Research Initiative‐Japan.

## CONFLICT OF INTEREST STATEMENT

All authors have no conflicts of interest to declare.

## Supporting information


Data S1:

## Data Availability

The datasets used and analyzed during the current study are available from the corresponding author upon reasonable request.

## References

[phy215774-bib-0001] Andrade‐Oliveira, V. , Amano, M. T. , Correa‐Costa, M. , Castoldi, A. , Felizardo, R. J. F. , de Almeida, D. C. , Bassi, E. J. , Moraes‐Vieira, P. M. , Hiyane, M. I. , Rodas, A. C. D. , Peron, J. P. S. , Aguiar, C. F. , Reis, M. A. , Ribeiro, W. R. , Valduga, C. J. , Curi, R. , Vinolo, M. A. R. , Ferreira, C. M. , & Câmara, N. O. S. (2015). Gut bacteria products prevent AKI induced by ischemia‐reperfusion. Journal of the American Society of Nephrology, 26(8), 1877–1888. 10.1681/ASN.2014030288 25589612 PMC4520159

[phy215774-bib-0002] Arai, H. , & Yanagita, M. (2020). Janus‐faced: Molecular mechanisms and versatile nature of renal fibrosis. Kidney360, 1(7), 697–704. 10.34067/kid.0001972020 35372942 PMC8815544

[phy215774-bib-0003] Aranda‐Rivera, A. K. , Cruz‐Gregorio, A. , Aparicio‐Trejo, O. E. , Ortega‐Lozano, A. J. , & Pedraza‐Chaverri, J. (2021). Redox signaling pathways in unilateral ureteral obstruction (UUO)‐induced renal fibrosis. Free Radical Biology and Medicine, 172, 65–81. 10.1016/j.freeradbiomed.2021.05.034 34077780

[phy215774-bib-0004] Bhargava, P. , & Schnellmann, R. G. (2017). Mitochondrial energetics in the kidney. Nature Reviews Nephrology, 13(10), 629–646. 10.1038/nrneph.2017.107 28804120 PMC5965678

[phy215774-bib-0005] Dan Dunn, J. , Alvarez, L. A. J. , Zhang, X. , & Soldati, T. (2015). Reactive oxygen species and mitochondria: A nexus of cellular homeostasis. Redox Biology, 6, 472–485. 10.1016/j.redox.2015.09.005 26432659 PMC4596921

[phy215774-bib-0006] Dong, W. , Jia, Y. , Liu, X. , Zhang, H. , Li, T. , Huang, W. , Chen, X. , Wang, F. , Sun, W. , & Wu, H. (2017). Sodium butyrate activates NRF2 to ameliorate diabetic nephropathy possibly via inhibition of HDAC. Journal of Endocrinology, 232(1), 71–83. 10.1530/JOE-16-0322 27799462

[phy215774-bib-0007] Frost, G. , Sleeth, M. L. , Sahuri‐Arisoylu, M. , Lizarbe, B. , Cerdan, S. , Brody, L. , Anastasovska, J. , Ghourab, S. , Hankir, M. , Zhang, S. , Carling, D. , Swann, J. R. , Gibson, G. , Viardot, A. , Morrison, D. , Louise Thomas, E. , & Bell, J. D. (2014). The short‐chain fatty acid acetate reduces appetite via a central homeostatic mechanism. Nature Communications, 5, 1–11. 10.1038/ncomms4611 PMC4015327 24781306

[phy215774-bib-0008] Go, A. S. , Chertow, G. M. , Fan, D. , McCulloch, C. E. , & Hsu, C. Y. (2004). Chronic kidney disease and the risks of death, cardiovascular events, and hospitalization. New England Journal of Medicine, 351(13), 1296–1305. 10.1056/NEJMoa041031 15385656

[phy215774-bib-0009] Heerspink, H. J. L. , Stefánsson, B. V. , Correa‐Rotter, R. , Chertow, G. M. , Greene, T. , Hou, F. F. , Mann, J. F. E. , McMurray, J. J. V. , Lindberg, M. , Rossing, P. , Sjöström, C. D. , Toto, R. D. , Langkilde, A. M. , & Wheeler, D. C. (2020). Dapagliflozin in patients with chronic kidney disease. New England Journal of Medicine, 383(15), 1436–1446. 10.1056/nejmoa2024816 32970396

[phy215774-bib-0010] Hu, S. , Kuwabara, R. , de Haan, B. J. , Smink, A. M. , & de Vos, P. (2020). Acetate and butyrate improve β‐cell metabolism and mitochondrial respiration under oxidative stress. International Journal of Molecular Sciences, 21(4), 1542. 10.3390/ijms21041542 32102422 PMC7073211

[phy215774-bib-0011] Huang, W. , Man, Y. , Gao, C. , Zhou, L. , Gu, J. , Xu, H. , Wan, Q. , Long, Y. , Chai, L. , Xu, Y. , & Xu, Y. (2020). Short‐chain fatty acids ameliorate diabetic nephropathy via GPR43‐mediated inhibition of oxidative stress and NF‐κB signaling. Oxidative Medicine and Cellular Longevity, 2020, 4074832. 10.1155/2020/4074832 32831998 PMC7422068

[phy215774-bib-0012] Irazabal, M. V. , & Torres, V. E. (2020). Reactive oxygen species and redox signaling in chronic kidney disease. Cell, 9(6), 1342. 10.7326/AITC201506020 PMC7349188 32481548

[phy215774-bib-0013] Jiang, H. , Shao, X. , Jia, S. , Qu, L. , Weng, C. , Shen, X. , Wang, Y. , Huang, H. , Wang, Y. , Wang, C. , Feng, S. , Wang, M. , Feng, H. , Geekiyanage, S. , Davidson, A. J. , & Chen, J. (2019). The mitochondria‐targeted metabolic tubular injury in diabetic kidney disease. Cellular Physiology and Biochemistry, 52(2), 156–171. 10.33594/000000011 30816665

[phy215774-bib-0014] Kalantar‐Zadeh, K. , Jafar, T. H. , Nitsch, D. , Neuen, B. L. , & Perkovic, V. (2021). Chronic kidney disease. The Lancet, 398(10302), 786–802. 10.1016/S0140-6736(21)00519-5 34175022

[phy215774-bib-0015] Li, C. , & Jackson, R. M. (2002). Reactive species mechanisms of cellular hypoxia‐reoxygenation injury. American Journal of Physiology‐Cell Physiology, 282, C227–C241. 10.1177/153857449803200307 11788333

[phy215774-bib-0016] Liu, M. , Ning, X. , Li, R. , Yang, Z. , Yang, X. , Sun, S. , & Qian, Q. (2017). Signalling pathways involved in hypoxia‐induced renal fibrosis. Journal of Cellular and Molecular Medicine, 21(7), 1248–1259. 10.1111/jcmm.13060 28097825 PMC5487923

[phy215774-bib-0017] MacHado, R. A. , Constantino, L. S. , Tomasi, C. D. , Rojas, H. A. , Vuolo, F. S. , Vitto, M. F. , Cesconetto, P. A. , de Souza, C. T. , Ritter, C. , & Dal‐Pizzol, F. (2012). Sodium butyrate decreases the activation of NF‐κB reducing inflammation and oxidative damage in the kidney of rats subjected to contrast‐induced nephropathy. Nephrology Dialysis Transplantation, 27(8), 3136–3140. 10.1093/ndt/gfr807 22273669

[phy215774-bib-0018] Martínez‐Reyes, I. , & Chandel, N. S. (2020). Mitochondrial TCA cycle metabolites control physiology and disease. Nature Communications, 11(1), 1–11. 10.1038/s41467-019-13668-3 PMC6941980 31900386

[phy215774-bib-0019] Mimura, I. , & Nangaku, M. (2010). The suffocating kidney: Tubulointerstitial hypoxia in end‐stage renal disease. Nature Reviews Nephrology, 6(11), 667–678. 10.1038/nrneph.2010.124 20877304

[phy215774-bib-0020] Mishima, E. , Fukuda, S. , Kanemitsu, Y. , Saigusa, D. , Mukawa, C. , Asaji, K. , Matsumoto, Y. , Tsukamoto, H. , Tachikawa, T. , Tsukimi, T. , Fukuda, N. N. , Ho, H. J. , Kikuchi, K. , Suzuki, C. , Nanto, F. , Suzuki, T. , Ito, S. , Soga, T. , Tomioka, Y. , & Abe, T. (2018). Canagliflozin reduces plasma uremic toxins and alters the intestinal microbiota composition in a chronic kidney disease mouse model. American Journal of Physiology‐Renal Physiology, 315(4), F824–F833. 10.1152/ajprenal.00314.2017 29167170

[phy215774-bib-0021] Mishima, E. , Fukuda, S. , Mukawa, C. , Yuri, A. , Kanemitsu, Y. , Matsumoto, Y. , Akiyama, Y. , Fukuda, N. N. , Tsukamoto, H. , Asaji, K. , Shima, H. , Kikuchi, K. , Suzuki, C. , Suzuki, T. , Tomioka, Y. , Soga, T. , Ito, S. , & Abe, T. (2017). Evaluation of the impact of gut microbiota on uremic solute accumulation by a CE‐TOFMS–based metabolomics approach. Kidney International, 92(3), 634–645. 10.1016/j.kint.2017.02.011 28396122

[phy215774-bib-0022] Müller, M. , Hernández, M. A. G. , Goossens, G. H. , Reijnders, D. , Holst, J. J. , Jocken, J. W. E. , van Eijk, H. , Canfora, E. E. , & Blaak, E. E. (2019). Circulating but not faecal short‐chain fatty acids are related to insulin sensitivity, lipolysis and GLP‐1 concentrations in humans. Scientific Reports, 9(1), 1–9. 10.1038/s41598-019-48775-0 31467327 PMC6715624

[phy215774-bib-0023] Muratsu‐Ikeda, S. , Nangaku, M. , Ikeda, Y. , Tanaka, T. , Wada, T. , & Inagi, R. (2012). Downregulation of miR‐205 modulates cell susceptibility to oxidative and endoplasmic reticulum stresses in renal tubular cells. PLoS ONE, 7(7), e41462. 10.1371/journal.pone.0041462 22859986 PMC3408438

[phy215774-bib-0024] Nangaku, M. (2006). Chronic hypoxia and tubulointerstitial injury: A final common pathway to end‐stage renal failure. Journal of the American Society of Nephrology, 17(1), 17–25. 10.1681/ASN.2005070757 16291837

[phy215774-bib-0025] Nath, K. A. (1992). Tubulointerstitial changes as a major determinant in the progression of renal damage. American Journal of Kidney Diseases, 20(1), 1–17. 1621674 10.1016/s0272-6386(12)80312-x

[phy215774-bib-0026] Olaniyi, K. S. , Amusa, O. A. , Akinnagbe, N. T. , Ajadi, I. O. , Ajadi, M. B. , Agunbiade, T. B. , & Michael, O. S. (2021). Acetate ameliorates nephrotoxicity in streptozotocin‐nicotinamide‐induced diabetic rats: Involvement of xanthine oxidase activity. Cytokine, 142, 155501. 10.1016/j.cyto.2021.155501 33775493

[phy215774-bib-0027] Panizo, S. , Martínez‐Arias, L. , Alonso‐Montes, C. , Cannata, P. , Martín‐Carro, B. , Fernández‐Martín, J. L. , Naves‐Díaz, M. , Carrillo‐López, N. , & Cannata‐Andía, J. B. (2021). Fibrosis in chronic kidney disease: Pathogenesis and consequences. International Journal of Molecular Sciences, 22(1), 1–19. 10.3390/ijms22010408 PMC7795409 33401711

[phy215774-bib-0028] Perkovic, V. , Jardine, M. J. , Neal, B. , Bompoint, S. , Heerspink, H. J. L. , Charytan, D. M. , Edwards, R. , Agarwal, R. , Bakris, G. , Bull, S. , Cannon, C. P. , Capuano, G. , Chu, P. L. , de Zeeuw, D. , Greene, T. , Levin, A. , Pollock, C. , Wheeler, D. C. , Yavin, Y. , … Mahaffey, K. W. (2019). Canagliflozin and renal outcomes in type 2 diabetes and nephropathy. New England Journal of Medicine, 380(24), 2295–2306. 10.1056/NEJMoa1811744 30990260

[phy215774-bib-0029] Perry, H. M. , Huang, L. , Wilson, R. J. , Bajwa, A. , Sesaki, H. , Yan, Z. , Rosin, D. L. , Kashatus, D. F. , & Okusa, M. D. (2018). Dynamin‐related protein 1 deficiency promotes recovery from AKI. Journal of the American Society of Nephrology, 29(1), 194–206. 10.1681/ASN.2017060659 29084809 PMC5748924

[phy215774-bib-0030] Pietrocola, F. , Galluzzi, L. , Bravo‐San Pedro, J. M. , Madeo, F. , & Kroemer, G. (2015). Acetyl coenzyme A: A central metabolite and second messenger. Cell Metabolism, 21(6), 805–821. 10.1016/j.cmet.2015.05.014 26039447

[phy215774-bib-0031] Pluznick, J. L. (2016). Gut microbiota in renal physiology: Focus on short‐chain fatty acids and their receptors. Kidney International, 90(6), 1191–1198. 10.1016/j.kint.2016.06.033 27575555 PMC5123942

[phy215774-bib-0032] Poll, B. G. , Xu, J. , Jun, S. , Sanchez, J. , Zaidman, N. A. , He, X. , Lester, L. , Berkowitz, D. E. , Paolocci, N. , Gao, W. D. , & Pluznick, J. L. (2021). Acetate, a short‐chain fatty acid, acutely lowers heart rate and cardiac contractility along with blood pressure. Journal of Pharmacology and Experimental Therapeutics, 377(1), 39–50. 10.1124/jpet.120.000187 33414131 PMC7985618

[phy215774-bib-0033] Sachse, A. , & Wolf, G. (2007). Angiotensin II‐induced reactive oxygen species and the kidney. Journal of the American Society of Nephrology, 18(9), 2439–2446. 10.1681/ASN.2007020149 17687073

[phy215774-bib-0034] Shi, L. , & Tu, B. P. (2015). Acetyl‐CoA and the regulation of metabolism: Mechanisms and consequences. Current Opinion in Cell Biology, 33, 125–131. 10.1016/J.CEB.2015.02.003 25703630 PMC4380630

[phy215774-bib-0035] Shubitowski, T. B. , Poll, B. G. , Natarajan, N. , & Pluznick, J. L. (2019). Short‐chain fatty acid delivery: Assessing exogenous administration of the microbiome metabolite acetate in mice. Physiological Reports, 7(4), 1–11. 10.14814/phy2.14005 PMC6391713 30810289

[phy215774-bib-0036] Sun, X. , Zhang, B. , Hong, X. , Zhang, X. , & Kong, X. (2013). Histone deacetylase inhibitor, sodium butyrate, attenuates gentamicin‐induced nephrotoxicity by increasing prohibitin protein expression in rats. European Journal of Pharmacology, 707(1–3), 147–154. 10.1016/j.ejphar.2013.03.018 23528351

[phy215774-bib-0037] Tahara, Y. , Yamazaki, M. , Sukigara, H. , Motohashi, H. , Sasaki, H. , Miyakawa, H. , Haraguchi, A. , Ikeda, Y. , Fukuda, S. , & Shibata, S. (2018). Gut microbiota‐derived short chain fatty acids induce circadian clock entrainment in mouse peripheral tissue. Scientific Reports, 8(1), 1–12. 10.1038/s41598-018-19836-7 29362450 PMC5780501

[phy215774-bib-0038] Trompette, A. , Gollwitzer, E. S. , Yadava, K. , Sichelstiel, A. K. , Sprenger, N. , Ngom‐Bru, C. , Blanchard, C. , Junt, T. , Nicod, L. P. , Harris, N. L. , & Marsland, B. J. (2014). Gut microbiota metabolism of dietary fiber influences allergic airway disease and hematopoiesis. Nature Medicine, 20(2), 159–166. 10.1038/nm.3444 24390308

[phy215774-bib-0039] Venegas, D. P. , de la Fuente, M. K. , Landskron, G. , González, M. J. , Quera, R. , Dijkstra, G. , Harmsen, H. J. M. , Faber, K. N. , & Hermoso, M. A. (2019). Short chain fatty acids (SCFAs)‐mediated gut epithelial and immune regulation and its relevance for inflammatory bowel diseases. Frontiers in Immunology, 10, 277. 10.3389/fimmu.2019.00277 30915065 PMC6421268

[phy215774-bib-0040] Wagenaar, C. A. , van de Put, M. , Bisschops, M. , Walrabenstein, W. , de Jonge, C. S. , Herrema, H. , & van Schaardenburg, D. (2021). The effect of dietary interventions on chronic inflammatory diseases in relation to the microbiome: A systematic review. Nutrients, 13(9), 3208. 10.3390/nu13093208 34579085 PMC8464906

[phy215774-bib-0042] Wong, J. M. W. , de Souza, R. , Kendall, C. W. C. , Emam, A. , & Jenkins, D. J. A. (2006). Colonic health: Fermentation and short chain fatty acids. Journal of Clinical Gastroenterology, 40(3), 235–243. 10.1097/00004836-200603000-00015 16633129

[phy215774-bib-0043] Xie, Y. , Bowe, B. , Mokdad, A. H. , Xian, H. , Yan, Y. , Li, T. , Maddukuri, G. , Tsai, C. Y. , Floyd, T. , & al‐Aly, Z. (2018). Analysis of the global burden of disease study highlights the global, regional, and national trends of chronic kidney disease epidemiology from 1990 to 2016. Kidney International, 94(3), 567–581. 10.1016/j.kint.2018.04.011 30078514

[phy215774-bib-0044] Xu, M. , Nagati, J. S. , Xie, J. , Li, J. , Walters, H. , Moon, Y. A. , Gerard, R. D. , Huang, C. L. , Comerford, S. A. , Hammer, R. E. , Horton, J. D. , Chen, R. , & Garcia, J. A. (2014). An acetate switch regulates stress erythropoiesis. Nature Medicine, 20(9), 1018–1026. 10.1038/nm.3587 PMC4159437 25108527

[phy215774-bib-0045] Yang, T. , Richards, E. M. , Pepine, C. J. , & Raizada, M. K. (2018). The gut microbiota and the brain–gut–kidney axis in hypertension and chronic kidney disease. Nature Reviews Nephrology, 14(7), 442–456. 10.1038/s41581-018-0018-2 29760448 PMC6385605

[phy215774-bib-0046] Yang, Y. , Zhang, G. , Yang, T. , Gan, J. , Xu, L. , & Yang, H. (2021). A flow‐cytometry‐based protocol for detection of mitochondrial ROS production under hypoxia. STAR Protocols, 2(2), 100466. 10.1016/j.xpro.2021.100466 33997804 PMC8086139

[phy215774-bib-0047] Youle, R. J. , & van der Bliek, A. M. (2012). Mitochondrial fission, fusion, and stress. Science, 337, 1062–1066. 22936770 10.1126/science.1219855 PMC4762028

[phy215774-bib-0048] Yu, C. , Zhang, M. , Liu, J. , Zhang, J. , Xu, J. , & Xu, W. (2022). Effects of sodium acetate on lipid metabolism, antioxidant capability and cell apoptosis of blunt snout bream (*Megalobrama amblycephala*) hepatocytes treated by sodium palmitate. Aquaculture Research, 53(3), 1098–1109. 10.1111/are.15651

[phy215774-bib-0049] Zhan, M. , Usman, I. M. , Sun, L. , & Kanwar, Y. S. (2015). Disruption of renal tubular mitochondrial quality control by Myo‐inositol oxygenase in diabetic kidney disease. Journal of the American Society of Nephrology, 26(6), 1304–1321. 10.1681/ASN.2014050457 25270067 PMC4446875

