# Supplementary material for: Acetate attenuates kidney fibrosis in an oxidative stress‐dependent manner
Source: Physiol Rep. 2023 Jul 18;11(14):e15774. doi: 10.14814/phy2.15774 (PMC10354006; doi:10.14814/phy2.15774)
Supplement: Supplementary file 1 — Data S1: [file PHY2-11-e15774-s001.pptx]

## Slide 1
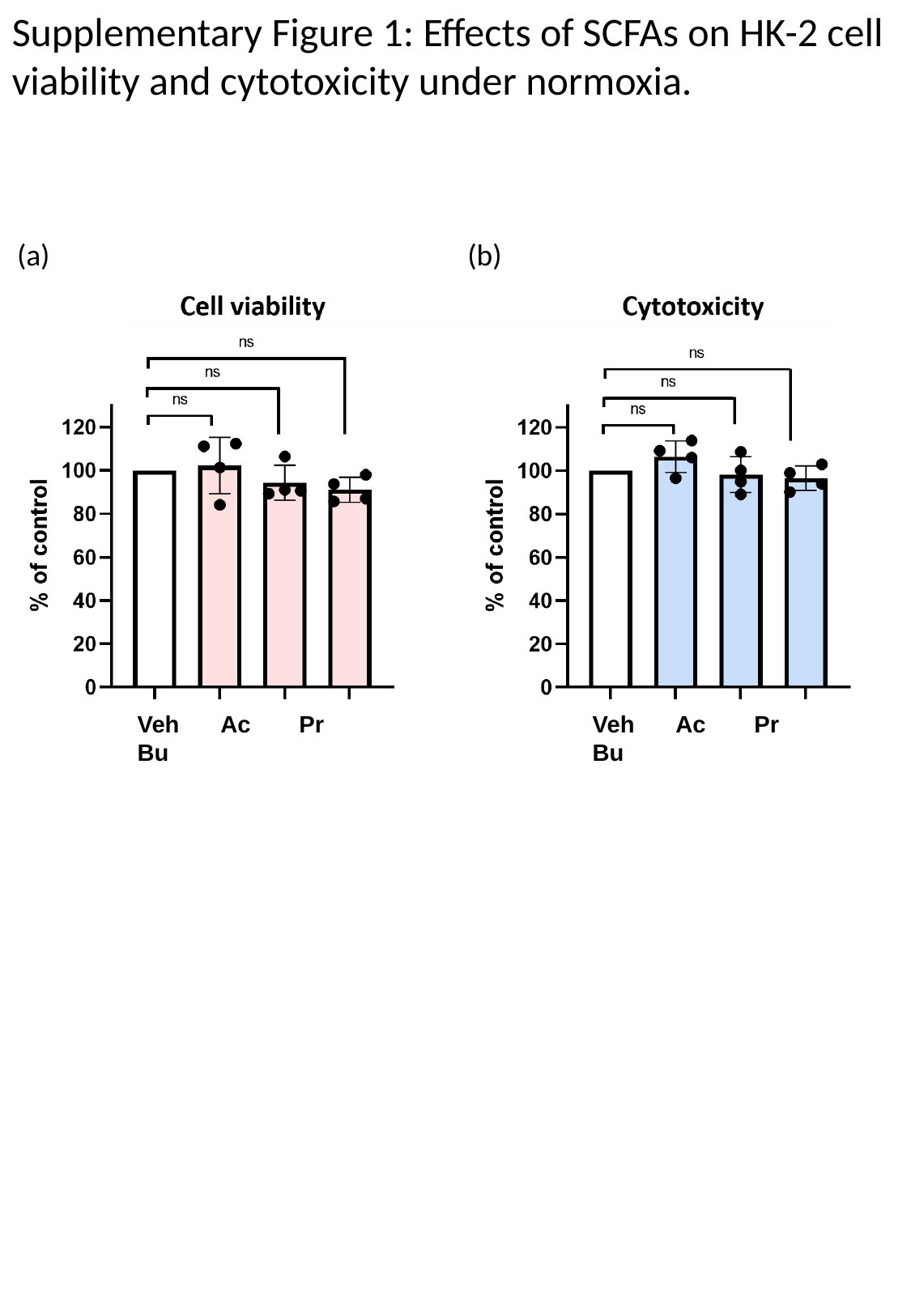

Supplementary Figure 1: Effects of SCFAs on HK-2 cell viability and cytotoxicity under normoxia.
(a)
(b)
Veh　 Ac 　Pr 　Bu
Veh　 Ac 　Pr 　Bu

## Slide 2
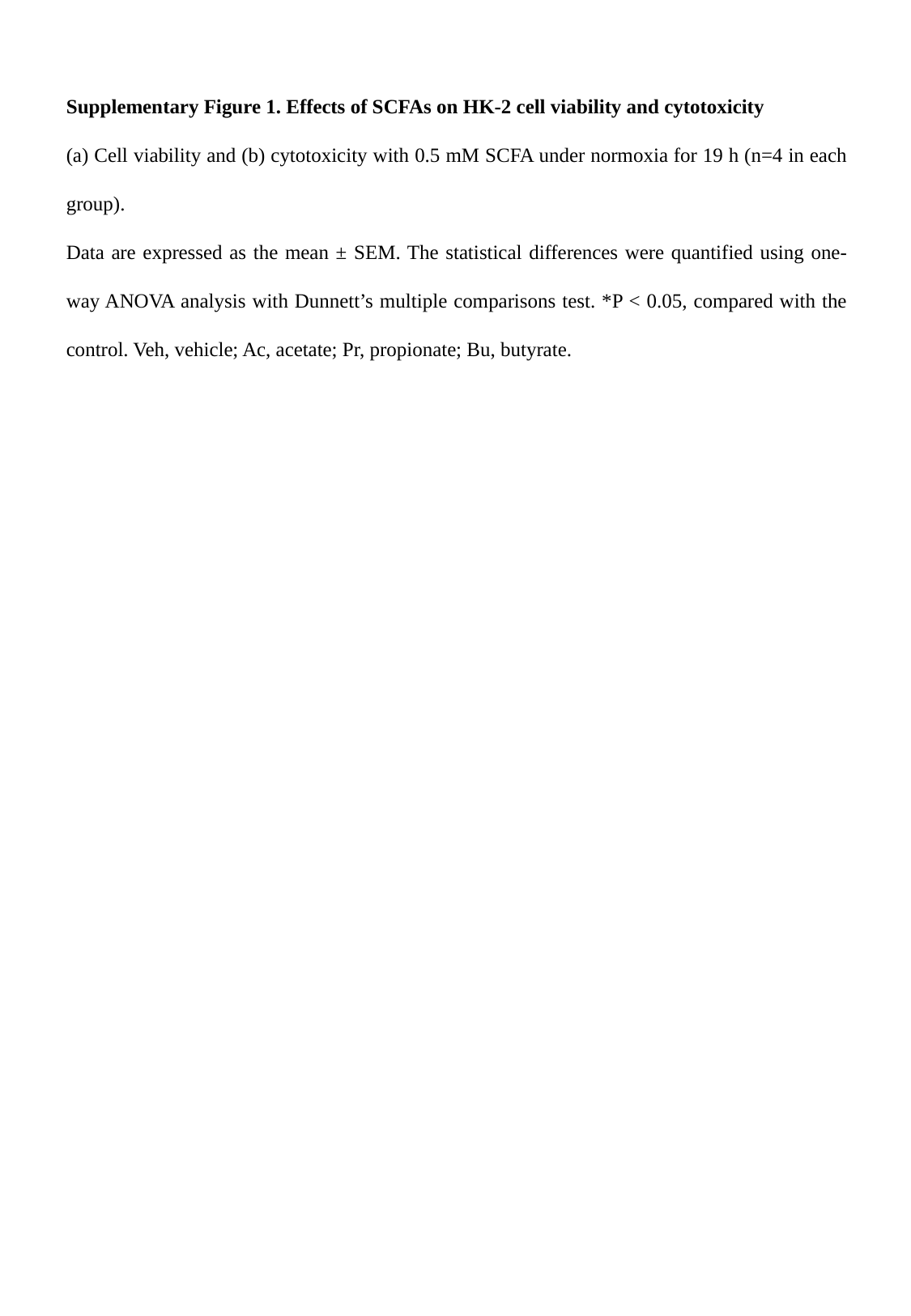

Supplementary Figure 1. Effects of SCFAs on HK-2 cell viability and cytotoxicity
(a) Cell viability and (b) cytotoxicity with 0.5 mM SCFA under normoxia for 19 h (n=4 in each group).
Data are expressed as the mean ± SEM. The statistical differences were quantified using one-way ANOVA analysis with Dunnett’s multiple comparisons test. *P < 0.05, compared with the control. Veh, vehicle; Ac, acetate; Pr, propionate; Bu, butyrate.

## Slide 3
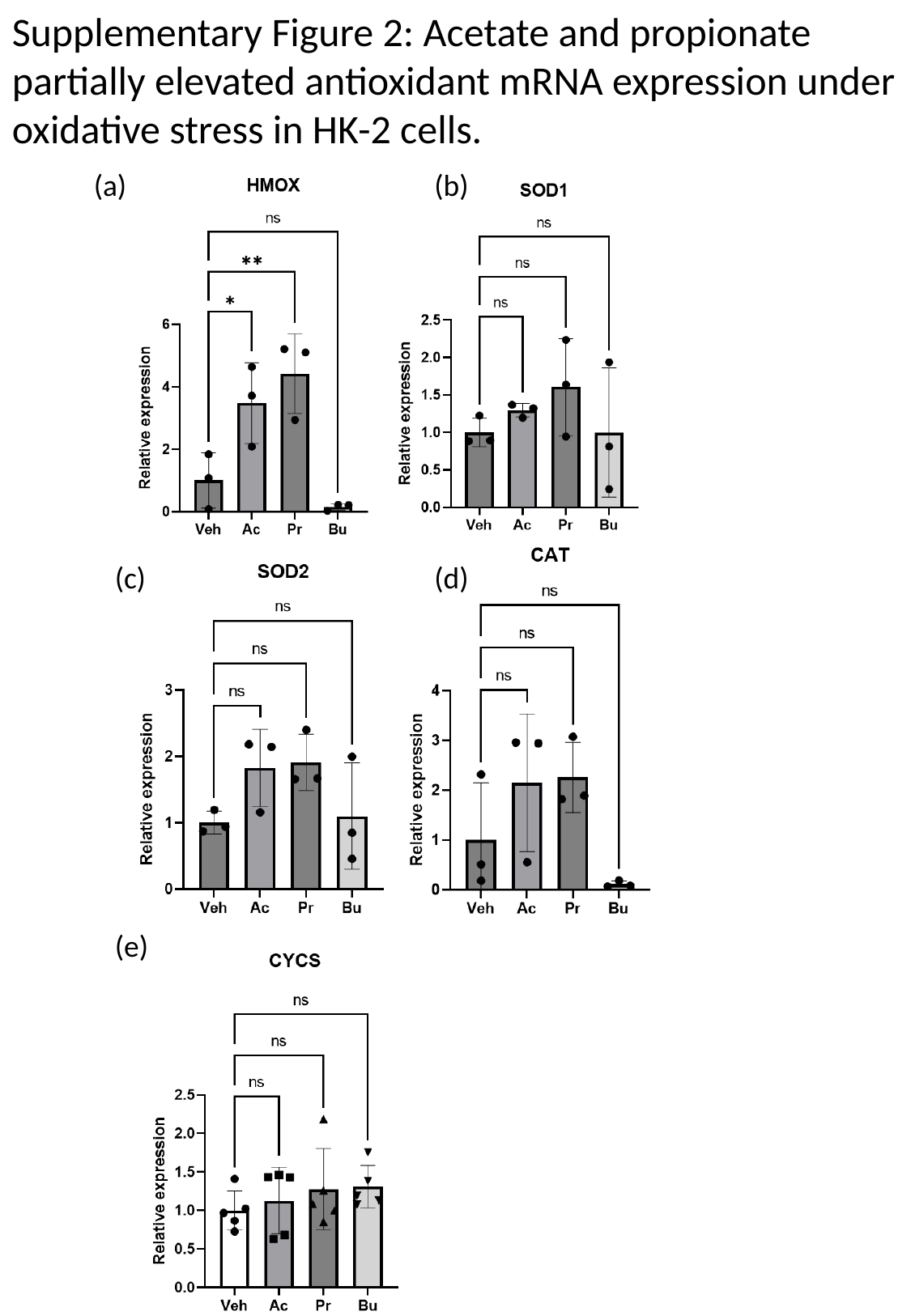

Supplementary Figure 2: Acetate and propionate partially elevated antioxidant mRNA expression under oxidative stress in HK-2 cells.
(a)
(b)
(c)
(d)
(e)

## Slide 4
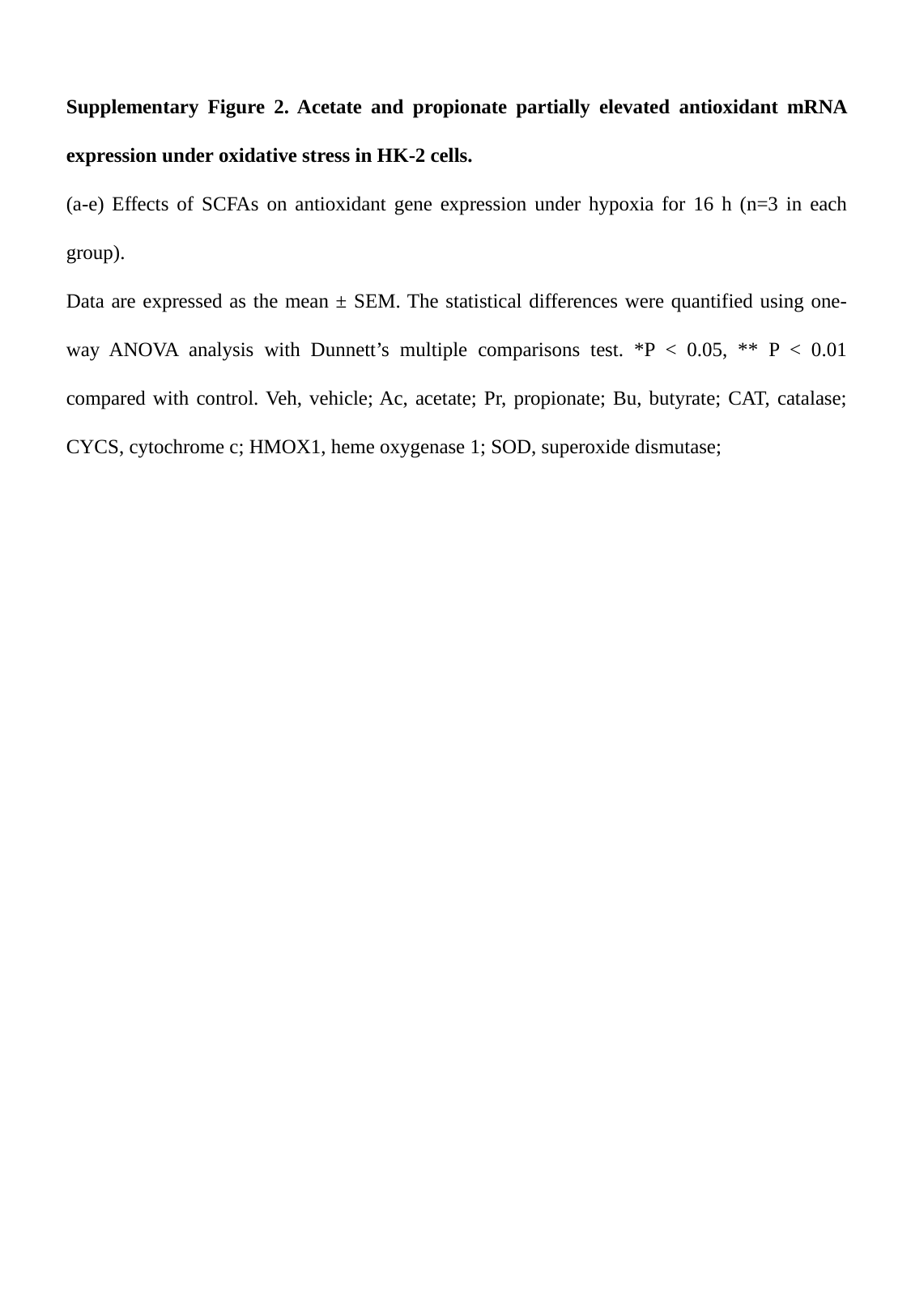

Supplementary Figure 2. Acetate and propionate partially elevated antioxidant mRNA expression under oxidative stress in HK-2 cells.
(a-e) Effects of SCFAs on antioxidant gene expression under hypoxia for 16 h (n=3 in each group).
Data are expressed as the mean ± SEM. The statistical differences were quantified using one-way ANOVA analysis with Dunnett’s multiple comparisons test. *P < 0.05, ** P < 0.01 compared with control. Veh, vehicle; Ac, acetate; Pr, propionate; Bu, butyrate; CAT, catalase; CYCS, cytochrome c; HMOX1, heme oxygenase 1; SOD, superoxide dismutase;

## Slide 5
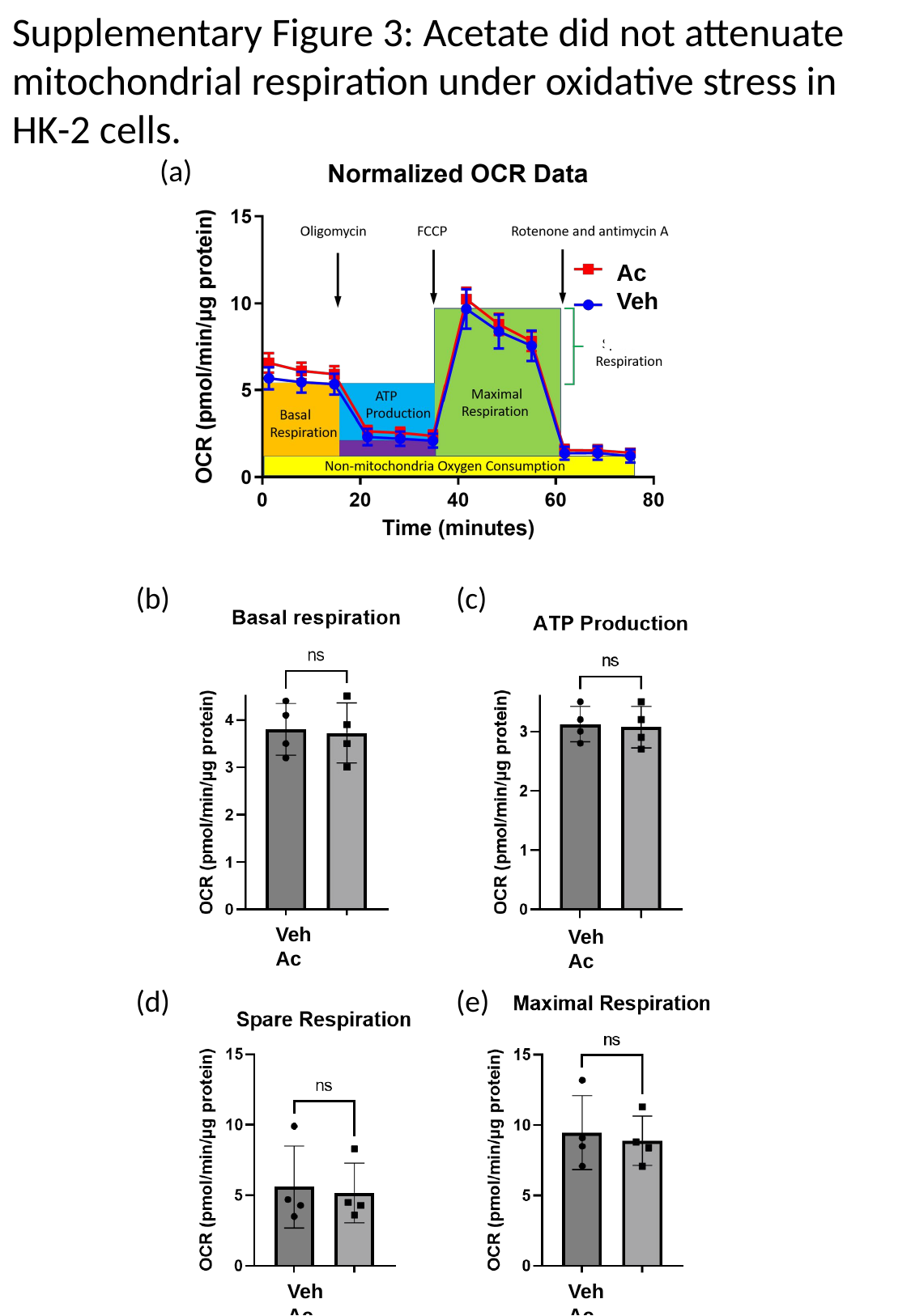

Supplementary Figure 3: Acetate did not attenuate mitochondrial respiration under oxidative stress in HK-2 cells.
(a)
Ac
Veh
(b)
(c)
Veh　 Ac
Veh　 Ac
(d)
(e)
Veh　 Ac
Veh　 Ac

## Slide 6
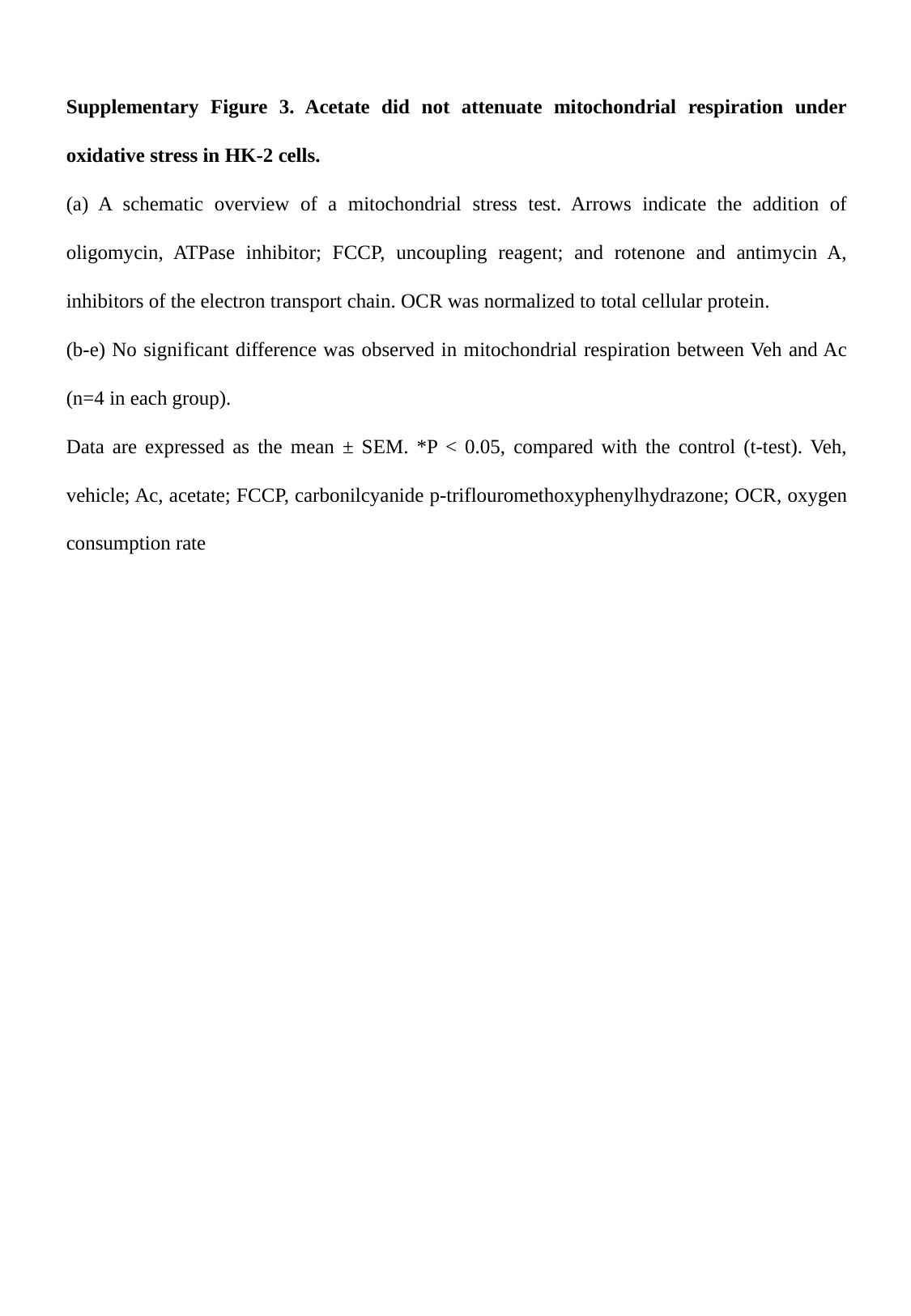

Supplementary Figure 3. Acetate did not attenuate mitochondrial respiration under oxidative stress in HK-2 cells.
(a) A schematic overview of a mitochondrial stress test. Arrows indicate the addition of oligomycin, ATPase inhibitor; FCCP, uncoupling reagent; and rotenone and antimycin A, inhibitors of the electron transport chain. OCR was normalized to total cellular protein.
(b-e) No significant difference was observed in mitochondrial respiration between Veh and Ac (n=4 in each group).
Data are expressed as the mean ± SEM. *P < 0.05, compared with the control (t-test). Veh, vehicle; Ac, acetate; FCCP, carbonilcyanide p-triflouromethoxyphenylhydrazone; OCR, oxygen consumption rate

## Slide 7
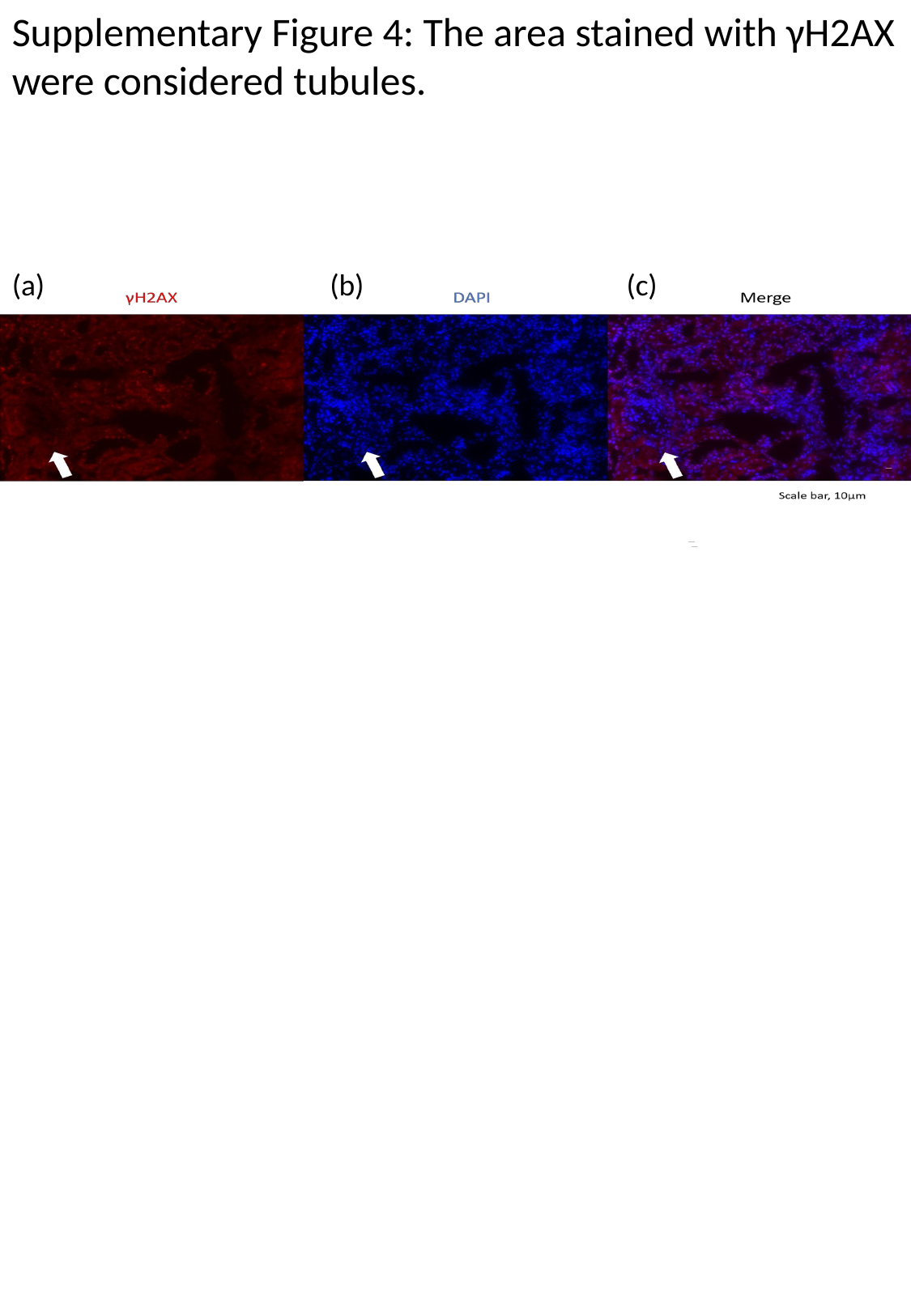

Supplementary Figure 4: The area stained with γH2AX were considered tubules.
(a)
(b)
(c)

## Slide 8
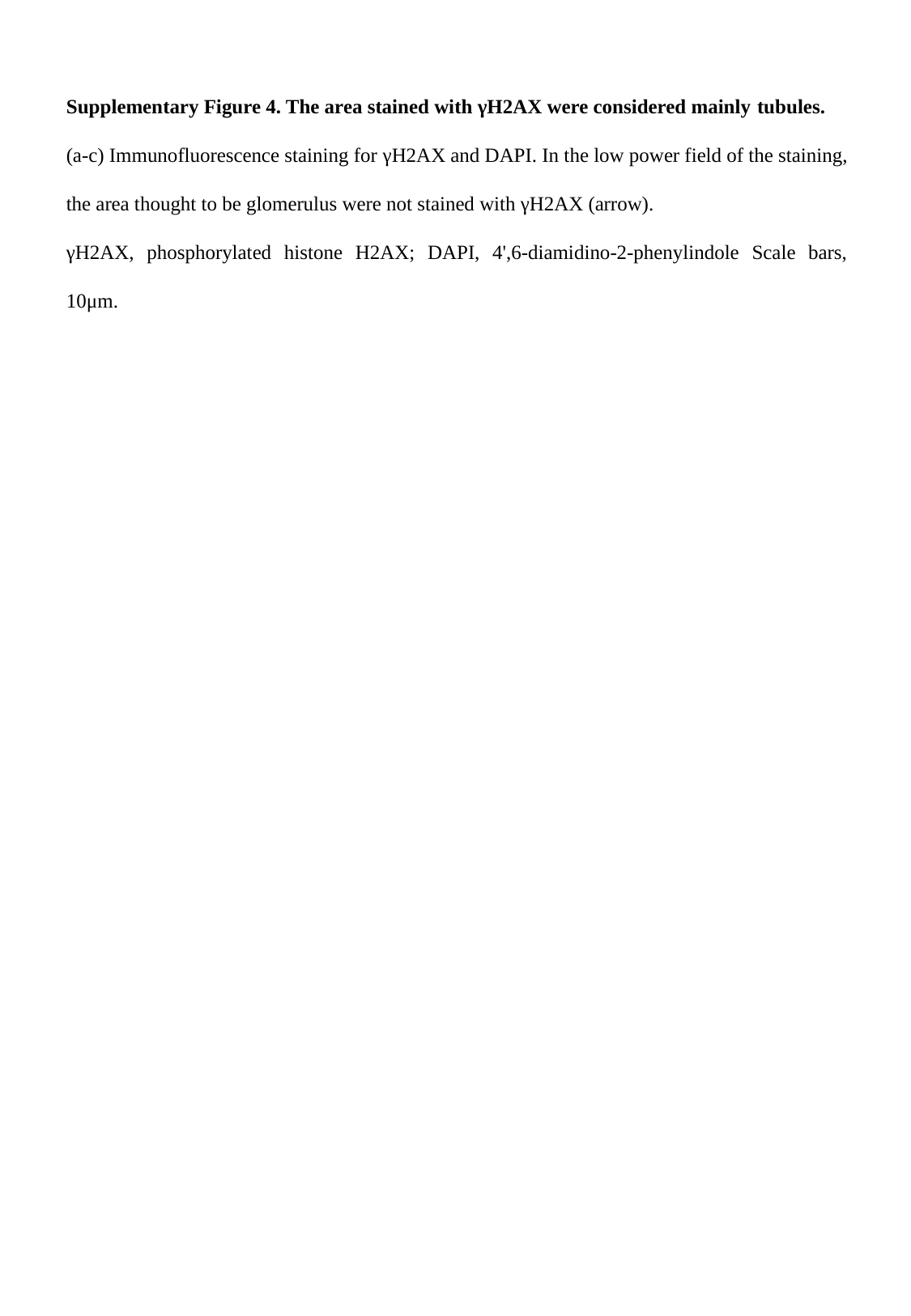

Supplementary Figure 4. The area stained with γH2AX were considered mainly tubules.
(a-c) Immunofluorescence staining for γH2AX and DAPI. In the low power field of the staining, the area thought to be glomerulus were not stained with γH2AX (arrow).
γH2AX, phosphorylated histone H2AX; DAPI, 4',6-diamidino-2-phenylindole Scale bars, 10μm.

## Slide 9
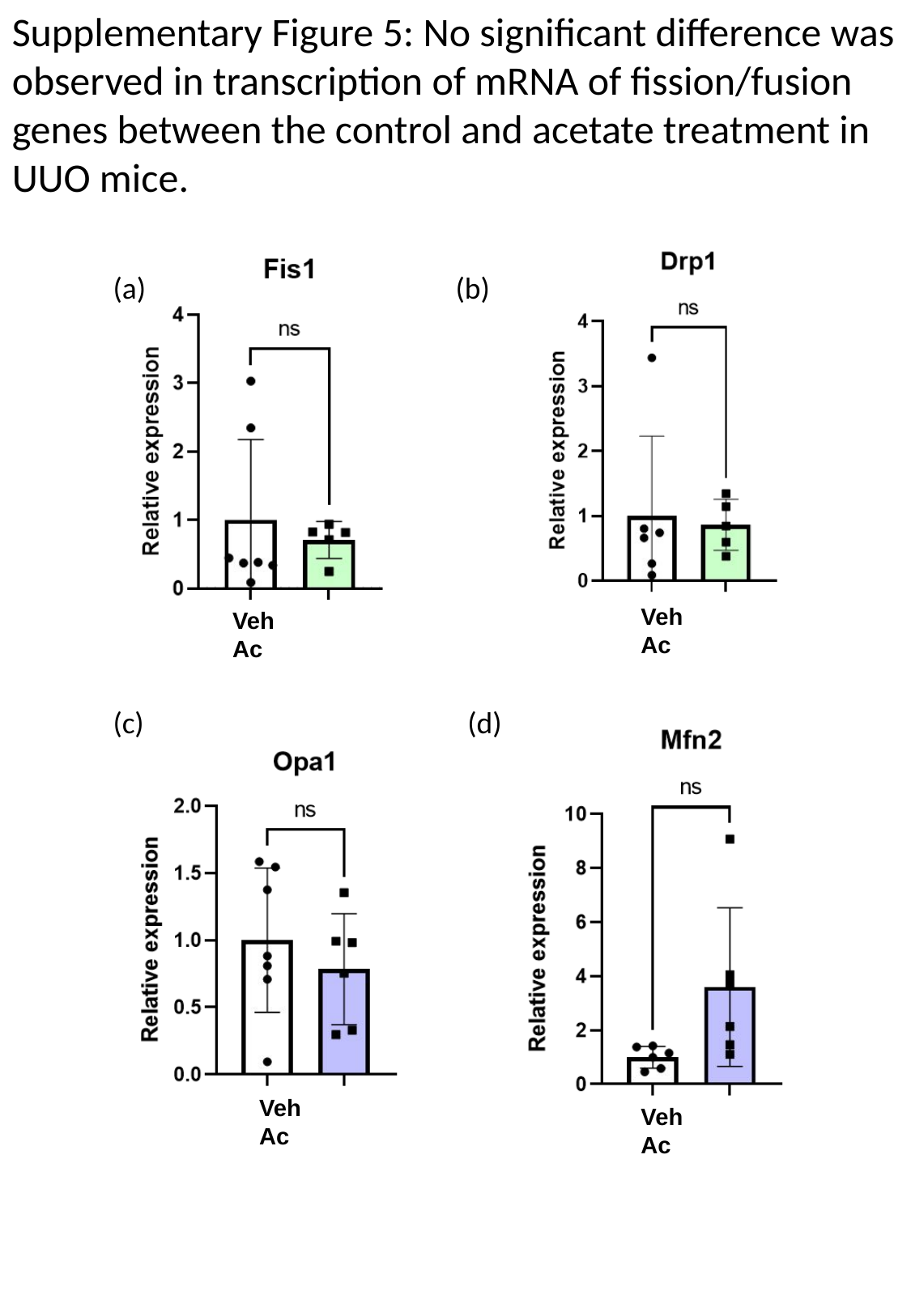

Supplementary Figure 5: No significant difference was observed in transcription of mRNA of fission/fusion genes between the control and acetate treatment in UUO mice.
Veh　 Ac
Veh　 Ac
(a)
(b)
(c)
(d)
Veh　 Ac
Veh　 Ac

## Slide 10
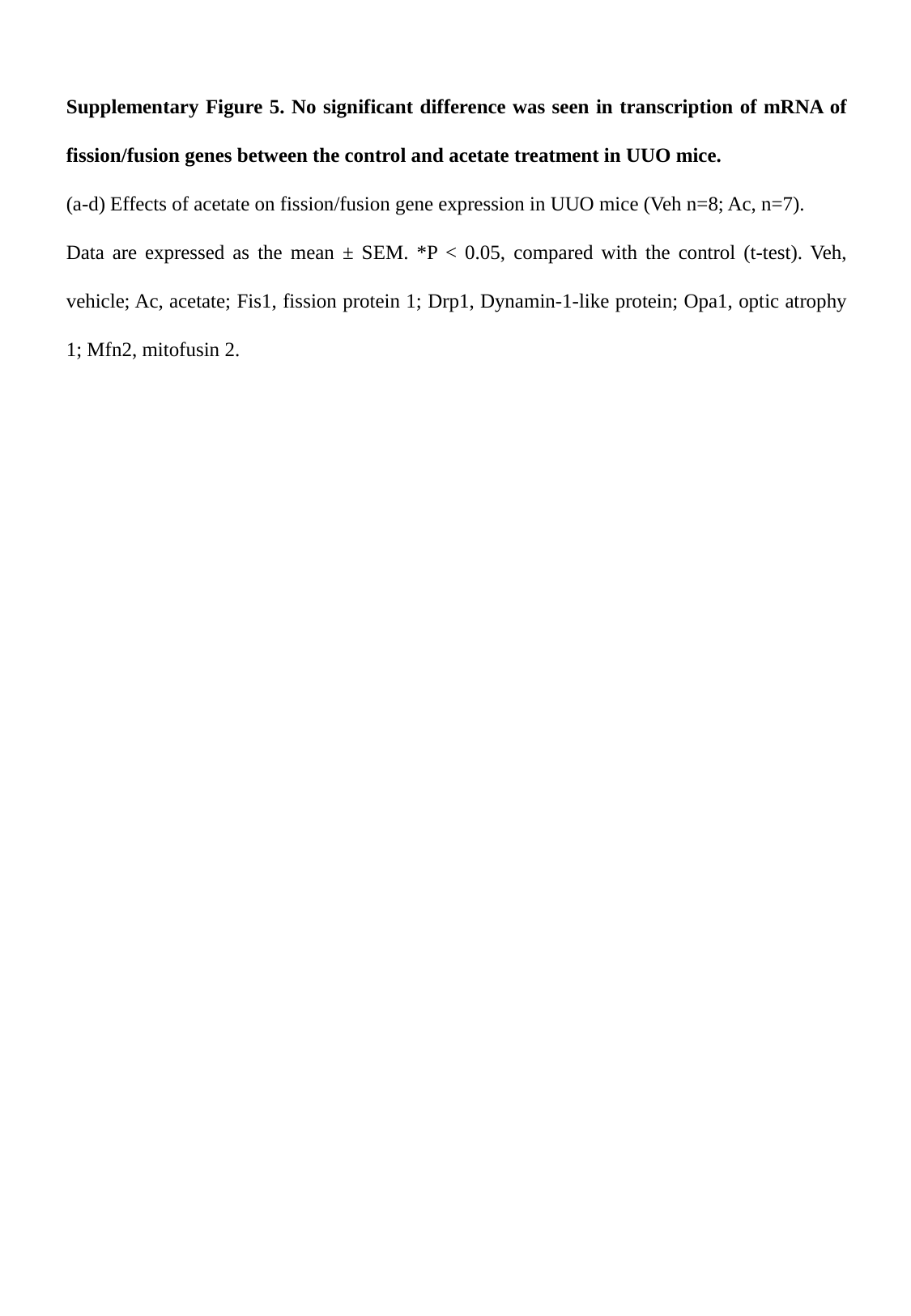

Supplementary Figure 5. No significant difference was seen in transcription of mRNA of fission/fusion genes between the control and acetate treatment in UUO mice.
(a-d) Effects of acetate on fission/fusion gene expression in UUO mice (Veh n=8; Ac, n=7).
Data are expressed as the mean ± SEM. *P < 0.05, compared with the control (t-test). Veh, vehicle; Ac, acetate; Fis1, fission protein 1; Drp1, Dynamin-1-like protein; Opa1, optic atrophy 1; Mfn2, mitofusin 2.
